# Functional Effects of ARV-1502 Analogs Against Bacterial Hsp70 and Implications for Antimicrobial Activity

**DOI:** 10.3389/fchem.2022.798006

**Published:** 2022-02-09

**Authors:** Alexandra Brakel, Lisa Kolano, Carl N. Kraus, Laszlo Otvos, Ralf Hoffmann

**Affiliations:** ^1^ Faculty of Chemistry and Mineralogy, Institute of Bioanalytical Chemistry, Universität Leipzig, Leipzig, Germany; ^2^ Center for Biotechnology and Biomedicine, Universität Leipzig, Leipzig, Germany; ^3^ Aceragen, Inc., Durham, NC, United States; ^4^ Institute of Medical Microbiology, Semmelweis University, Budapest, Hungary

**Keywords:** proline-rich antimicrobial peptide (PrAMP), Chex1-Arg20, 70 kDa heat shock protein DnaK, co-chaperones DnaJ and GrpE, ATPase activity, refolding, *Escherichia coli*, *Staphylococcus aureus*

## Abstract

The antimicrobial peptide (AMP) ARV-1502 was designed based on naturally occurring short proline-rich AMPs, including pyrrhocoricin and drosocin. Identification of chaperone DnaK as a therapeutic target in *Escherichia coli* triggered intense research on the ligand-DnaK-interactions using fluorescence polarization and X-ray crystallography to reveal the binding motif and characterize the influence of the chaperone on protein refolding activity, especially in stress situations. In continuation of this research, 182 analogs of ARV-1502 were designed by substituting residues involved in antimicrobial activity against Gram-negative pathogens. The peptides synthesized on solid-phase were examined for their binding to *E. coli* and *S. aureus* DnaK providing 15 analogs with improved binding characteristics for at least one DnaK. These 15 analogs were distinguished from the original sequence by their increased hydrophobicity parameters. Additionally, the influence of the entire DnaK chaperone system, including co-chaperones DnaJ and GrpE on refolding and ATPase activity, was investigated. The increasingly hydrophobic peptides showed a stronger inhibitory effect on the refolding activity of *E. coli* chaperones, reducing protein refolding by up to 64%. However, these more hydrophobic peptides had only a minor effect on the ATPase activity. The most dramatic changes on the ATPase activity involved peptides with aspartate substitutions. Interestingly, these peptides resulted in a 59% reduction of the ATPase activity in the *E. coli* chaperone system whereas they stimulated the ATPase activity in the *S. aureus* system up to 220%. Of particular note is the improvement of the antimicrobial activity against *S. aureus* from originally >128 µg/mL to as low as 16 µg/mL. Only a single analog exhibited improved activity over the original value of 8 µg/mL against *E. coli*. Overall, the various moderate-throughput screenings established here allowed identifying (un)favored substitutions on 1) DnaK binding, 2) the ATPase activity of DnaK, 3) the refolding activity of DnaK alone or together with co-chaperones, and 4) the antimicrobial activity against both *E. coli* and *S. aureus*.

## 1 Introduction

Proline-rich antimicrobial peptides (PrAMP) have been intensively studied with special emphasis on their mode of action, protease stability, and toxicity (Scocchi et al., 2011; Hansen et al., 2012; Li et al., 2014; Böttger et al., 2017). Peptide Chex1-Arg20 (recently termed ARV-1502; Chex-RPDKPRPYLPRPRPPRPVR-NH_2_; Chex denotes 1-amino-cyclohexane carboxylic acid) and its dimer A3-APO were designed based on a sequence comparison of different naturally occurring PrAMPs, such as pyrrhocoricin and drosocin (Otvos et al., 2005; Noto et al., 2008), to obtain analogs with improved preclinical properties against various infections. In diverse studies, ranging from MICs to infection models and finally pharmacokinetics, ARV-1502 proved to be a very promising candidate against infections induced by Enterobacteriaceae (Ostorhazi et al., 2017; Otvos et al., 2018; Brakel et al., 2019). Its binding to the bacterial chaperone DnaK, which was first identified as the lethal target but is currently considered as a secondary, non-lethal, target after the 70S ribosome, was thoroughly investigated (Zahn et al., 2013). ARV-1502 binds *via* residues YLPRP to the hydrophobic pocket of the 27 kDa C-terminal substrate binding domain (SBD) of DnaK influencing its functional activity (Zahn et al., 2013). These residues are also involved in the *in vitro* antimicrobial activity of pyrrhocoricin analogs (Cassone et al., 2008). DnaK belongs to the highly conserved family of 70 kDa heat shock proteins and acts as a molecular chaperone, supporting protein folding, especially in stress conditions (Mayer and Bukau, 2005). DnaK is an ATP-dependent enzyme, with the ATPase activity located in the 42 kDa N-terminal nucleotide binding domain (NBD) (Szabo et al., 1994). The intrinsic ATPase activity of DnaK is low, but it can be accelerated *in vitro* by co-chaperones DnaJ and GrpE (Chang et al., 2008). The rate-limiting step is the release of the ADP generated after hydrolysis of ATP, whereby DnaK transitions from the high (ADP-bound) to the low peptide affinity state (ATP-bound). The nucleotide exchange factor GrpE supports this step initiating a new cycle (Harrison and Kuriyan, 1997; Brehmer et al., 2001), which starts with Hsp40 chaperone transferring a peptide or a protein to the opened SBD of DnaK (Han and Christen, 2003). Since both DnaK and DnaJ prefer hydrophobic protein and peptide sequences, especially sequences containing leucine and phenylalanine, DnaJ is able to pre-select the substrates (Brehmer et al., 2001). In addition, the J-domain of DnaJ stimulates the ATP hydrolysis of DnaK by binding to its NBD (Karzai and McMacken, 1996). Due to the allosteric coupling of NBD and SBD, the C-terminal lid closes initiating a proper folding of the protein. Unlike ATP hydrolysis, protein folding requires the presence of co-chaperones (Mayer and Bukau, 2005). The interaction between the 70 kDa chaperone and its co-chaperones has been extensively studied in recent years using the *E. coli* DnaK system (Li et al., 2016). *E. coli* DnaK exhibits high homology to the 70 kDa chaperones of other organisms including mammals and humans (Winardhi et al., 2018). As the human chaperone Hsp70 is also associated with misfolding diseases (e.g., Huntington’s disease) and cancer, it appears to be a promising pharmaceutical target to inhibit the cellular chaperone machinery (Mosser and Morimoto, 2004; Schlecht et al., 2013). Molecular insights into the substrate-target interactions are very useful for a rational inhibitor design. PrAMPs are excellent DnaK substrates and are additionally interesting drug options due to their good antimicrobial and host defense activities. This study describes the influence of PrAMP ARV-1502 and 182 substituted analogs on the chaperone system of the Gram-negative bacterium *E. coli* and the Gram-positive bacterium *S. aureus* to identify amino acid substitutions affecting the functional properties of different DnaK alleles. Moreover, the relationship between the influence on the protein refolding activity and the antimicrobial activity of the novel peptides was thoroughly examined.

## Materials and Methods

### Reagents

Ammonium heptamolybdate tetrahydrate (>99%), dithiothreitol (DTT, >99%), glycerol (>99.5%), LB-broth, d-luciferin sodium salt (>99%), lysozyme (≥45,000 FIP U/mg), malachite green oxalate, sodium chloride (>99.5%) and sodium dodecyl sulfate (SDS, >99.5%) were purchased from Carl Roth GmbH and Co. KG (Karlsruhe, Germany). Adenosine 5′-triphosphate disodium salt hydrate (ATP, >99%), antifoam Y-30 emulsion, coenzyme A (free acid), disodium hydrogen phosphate (>98%), imidazole (>99.5%), luciferase (from *Photinus pyralis*, recombinant), magnesium acetate tetrahydrate (>99%), magnesium sulfate (>97%), potassium chloride (>99%), potassium dihydrogen phosphate (>98%), sodium acetate (>99%), sulfuric acid (>95%) and thiazolyl blue tetrazolium bromide (MTT; ≥97.5%) were obtained from Sigma Aldrich Chemie GmbH (Taufkirchen, Germany). cOmplete™ Mini EDTA-free protease inhibitor cocktail and DNase I (from bovine pancreas) were purchased from Roche Deutschland Holding GmbH (Mannheim, Germany). Magnesium chloride hexahydrate (>99%) and β-mercaptoethanol were obtained from Fluka (Buchs, Germany). EDTA (>99%), HEPES (>99.5%), and Tris ultrapure (>99.9%) were purchased from AppliChem GmbH (Darmstadt, Germany). Casein (from bovine milk) and guanidinium hydrochloride (>99.9%) were obtained from CalbioChem™ (San Diego, CA, United States). Ammonium persulfate, acrylamide/bis solution (30% w/v), Coomassie Brilliant blue G250, and TEV protease (10 U/µl) were purchased from SERVA electrophoresis GmbH (Heidelberg, Germany). Gibco^®^ DMEM/F-12 medium, Gibco^®^ PBS, Gibco^®^ Penicillin-Streptomycin (10,000 U/mL) and Gibco^®^ Trypsin-EDTA (0.5%) were obtained from Life Technologies GmbH (Darmstadt, Germany). Acetonitrile (ULC-MS grade, >99.97%) and formic acid (ULC-MS grade, >99%) were purchased from Biosolve B.V. (Valkenswaald, Netherlands).

Water (resistance R > 18 mΩ/cm; total organic content <10 ppb) was purified by a PureLab Ultra Analytic system (ELGA Lab Water, Celle, Germany).

### Peptide Synthesis

ARV-1502 acetate was obtained from PolyPeptide Laboratories (SanDiego, CA, United States) as white powder with a purity of 97.3% according to RP-HPLC. The residual TFA content was 0.05%. The identity was further confirmed by amino acid analysis (Asx, Pro, Val, Leu, Tyr, Lys, and Arg).

The 182 substituted analogs of ARV-1502 were obtained from ABclonal, Inc. (Woburn, United States). These peptides were purified by RP-HPLC using an acetonitrile gradient in the presence of 0.1% TFA. Masses were confirmed by ESI-MS and the purities (>80%) were determined by RP-HPLC recording the absorbance at 214 nm.

Peptides containing a N-terminal 5 (6)-carboxyfluorescein-label were synthesized in-house by Fmoc/^t^Bu-chemistry on Rink amide resin and purified by RP-HPLC using an acetonitrile gradient in the presence of 0.1% TFA. Masses were confirmed by ESI-MS and the purities (>95%) were determined by RP-HPLC recording the absorbance at 214 nm.

### Protein Expression and Purification

DnaK (UniProt-ID P0A6Y8 and P99110), DnaJ (UniProt-ID P08622 and P63971), and GrpE (UniProt-ID P09372 and P99086, downloaded on 26.08.2021), from *Escherichia coli* and *Staphylococcus aureus* were overexpressed in *E. coli* (DE3) Rosetta pLysS after induction with IPTG (Zahn et al., 2013). Briefly, the coding sequences were cloned into a pET 15b vector (GeneScript Biotech BV, Leiden, Netherlands) using restriction enzymes NdeI and NcoI. *E. coli* (DE3) Rosetta pLysS was grown in LB broth containing ampicillin (0.1 g/L), chloramphenicol (34 mg/L), and 0.0075% (v/v) Antifoam Y-30 to reach an optical density of 0.6 recorded at 600 nm on an orbital shaker (180 rpm, 30°C). Expression was induced with IPTG (1 mmol/L). After 4 h, the cells were harvested, resuspended in lysis buffer (20 mmol/L KH_2_PO_4_/Na_2_HPO_4_, 0.5 mol/L NaCl, 30 mmol/L imidazole, 5% (v/v) glycerol) containing 2 mmol/L DTT for DnaK or 3 mmol/L DTT for DnaJ expression (pH 8.0), and disrupted by FastPrep-24™ 5G (60 s, 4 m/s, three cycles, MP Biomedicals Germany GmbH, Eschwege, Germany). Proteins were purified using immobilized metal affinity chromatography (IMAC, HisTrap™ HP, GE Healthcare Bio-Sciences AB, Uppsala, Sweden). The N-terminal sequence MGSSHHHHHHSSGENLYFQ was cleaved with TEV protease overnight at room temperature leaving only the sequence GGTHT at the N-terminus of all six proteins. The TEV protease, the cleaved His-tag, and proteins with the remaining His-tag were removed by IMAC. Proteins were stored at −80°C in Tris-HCl (20 mmol/L), KCl (150 mmol/L), MgCl_2_ (5 mmol/L), and glycerol (5%, v/v) containing 2 mmol/L DTT (only DnaK) or 3 mmol/L DTT (only DnaJ) at pH 7.5. The purity of all proteins was verified with SDS-PAGE. The corresponding bands were excised, incubated with trypsin (in-gel digest), and the protein confirmed by identifying the tryptic peptides by LC-MS.

### Fluorescence Polarization Assays

Dissociation (K_d_) and inhibitory constants (K_i_) were measured using a previously reported protocol with slight modifications (Krizsan et al., 2015; Kolano et al., 2020). Briefly, black 384-well plates (flat bottom, Greiner Bio-One GmbH, Frickenhausen, Germany) were blocked with 0.5% (w/v) casein in phosphate buffered saline (PBS; 10 mmol/L Na_2_HPO_4_, 0.2 mmol/L KH_2_PO_4_, 137 mmol/L NaCl, 2.7 mmol/L KCl, pH 7.4) containing 0.05% (w/v) Tween^®^ 20 (PBST) at 4°C overnight and washed three times with PBST. K_d_ values were measured by dissolving the proteins in FP-buffer (20 mmol/L Tris-HCl, 150 mmol/L KCl, 5 mmol/L MgCl_2_, 1 mmol/L NaN_3_, 2 mmol/L DTT, pH 7.5) and serially diluting them twofold in 23 steps on the plate (20 µL/well). The 5 (6)-carboxyfluorescein-labeled peptide was added (20 μL/well, 40 nmol/L), incubated (2 h, 28°C, dark), and the fluorescence polarization recorded at an excitation wavelength (*λ*_ex_) of 485 nm and an emission wavelength (λ_em_) of 535 nm using a PARADIGM™ microplate reader (Beckman Coulter, Krefeld, Germany). K_i_ values were determined using a twofold dilution series of unlabeled peptide (20 µL/well) and DnaK (10 µL/well). The plate was incubated at 28°C in the dark for 90 min before the Cf-labeled peptides were added (10 µL/well, 80 nmol/L). After a second incubation (90 min, 28°C), the fluorescence polarization was recorded (*λ*_ex_ = 485 nm, *λ*_em_ = 535 nm). Dissociation and inhibitory constants were calculated by fitting the data with a variable slope parameter [y = min + (max- min)/(1+(x/K_d_) ^−Hill slope^)] using SigmaPlot 13 (Systat Software Inc., San Jose, CA, United States).

The peptide binding screening with *E. coli* or *S. aureus* DnaK used unlabeled peptide (10 µmol/L for *E. coli*, 70 µmol/L for *S. aureus*; 20 µL/well) pipetted into a 384-well plate and DnaK solutions of 20 µmol/L (*E. coli*) or 64 µmol/L (*S. aureus*; 10 µL/well). The plate was incubated (90 min, 28°C, dark) and Cf-ARV-1502 (80 nmol/L; 10 µL/well) added. After a second incubation (90 min, 28°C, dark), the fluorescence polarization was recorded (*λ*_ex_ = 485 nm, *λ*_em_ = 535 nm) on the PARADIGM™ microplate reader. Each peptide was screened in duplicate. Control samples either lacked the unlabeled peptide (maximum) or contained Cf-ARV-1502 in buffer (minimum).

### ATPase Activity Assay

ATPase activity of *E. coli* and *S. aureus* chaperones was studied in the presence of an AMP using a 384-well plate colorimetric assay (Chang et al., 2008; Zahn et al., 2013). The peptide screening used a chaperone mix of DnaK, DnaJ, and GrpE (final concentrations of 0.6, 1.0, and 0.9 µmol/L, respectively) dissolved in assay buffer (20 mmol/L Tris-HCl, 150 mmol/L KCl, 5 mmol/L MgCl_2_, pH 7.5). The chaperone mix (13.5 µL) was incubated with an aqueous peptide solution (1.5 µL; final concentration 0.3 mmol/L) at 37°C for 30 min in a non-binding 384-well plate (flat bottom, Greiner Bio-One GmbH). Each sample was prepared in triplicates on the same plate. The reaction was started by addition of ATP dissolved in assay buffer (5 µL, 4 mmol/L) and the plate centrifuged (500 × g, 2 min; Allegra™ 21R, Beckmann Coulter, Krefeld, Germany) before incubated (37°C, 2 h). The released phosphate was quantified using an external phosphate dilution series (10–200 µmol/L KH_2_PO_4_ in assay buffer) prepared on each plate and treated equally as the samples. The reaction was stopped by adding assay buffer (80 µL) to all samples except the phosphate dilution series. Aliquots of all samples (10 µL) were transferred to a clear 384-well plate (flat bottom, Greiner Bio-One GmbH) and malachite green reagent added (90 µL; mixture of malachite green in water (0.04%, w/v), ammonium molybdate (2%, w/w) in aqueous sulfuric acid (3.5 mol/L), and water at a ratio of 1:1:3.6, v/v/v). After 15 min, the absorbance was recorded at 620 nm on the PARADIGM™ microplate reader. The phosphate standard curve was fitted by a hyperbolic equation y = (ax)/(b + x) + c.

### Denatured Luciferase Refolding Assay

Recombinant firefly luciferase (0.5 g/L) was denatured in luciferase buffer (25 mmol/L HEPES (pH 7.2), 50 mmol/L potassium acetate, 5 mmol/L DTT) containing guanidine hydrochloride (6 mol/L) at room temperature for 1 h (Wisén and Gestwicki, 2008). The denatured luciferase was 100fold diluted in luciferase buffer and incubated on ice for 20 min in the dark. Peptides (4 µL; final concentration 150 µmol/L) were incubated in a non-binding 384-well plate (flat bottom, Greiner Bio-One GmbH) with chaperone mix (41 µL; final concentrations: 240 nmol/L DnaK, 48 nmol/L DnaJ and 24 nmol/L GrpE) in refolding buffer [28 mmol/L HEPES (pH 7.6), 120 mmol/L potassium acetate, 12 mmol/L magnesium acetate, 2 mmol/L DTT, 0.5 mmol/L ATP, 8.8 mmol/L creatine phosphate, 35 U/mL creatine kinase) at 37°C for 30 min. The reaction was started by addition of denatured luciferase (5 µL). The plate was incubated (37°C, 2 h, dark) before 10 µL of each sample were transferred to a white 384-well plate (Greiner Bio-One GmbH) and diluted with detection buffer (20 µL; 25 mmol/L HEPES (pH 7.8), 8 mmol/L magnesium sulfate, 12 mmol/L DTT, 0.5 mmol/L ATP, 120 µmol/L coenzyme A, 100 µmol/L d-luciferin). After incubation (30°C, 10 min) in the PARADIGM™ microplate reader, the luminescence was recorded using an integration time of 0.5 s.

### Antimicrobial Activity

Minimal inhibitory concentrations (MICs) were determined using a liquid broth micro dilution assay in sterile 96-well plates (polystyrene F-bottom, Greiner Bio-One GmbH) and a total volume of 100 μL per well. Aqueous peptide solutions (10 g/L) were serially twofold diluted in 25% Mueller-Hinton broth 2 (25% MBH2) starting at a peptide concentration of 128–1 µg/mL (50 μL per well). Overnight cultures of bacteria, i.e., *E. coli* BW25113 or *S. aureus* DSM 6247, grown in 25% MHB2 were diluted 30-fold in 25% MHB2. After an incubation period of 4 h (37°C, 200 rpm), cells were diluted to 1.5 × 10^7^ cfu/mL, based on a McFarland test, and 50 μL were added to each well (final concentration of 7.5 × 10^6^ cfu/mL per well). The plates were incubated (37°C, 20 h) and the optical density was determined at 595 nm using the PARADIGM™ microplate reader. The MIC was defined as the lowest peptide concentration preventing visible bacterial growth.

### Cytotoxicity

Human embryonic kidney (HEK293) and human hepatoma (HepG2) cells were cultured in Dulbecco’s modified Eagle’s/Ham’s F-12 medium (DMEM/F-12) containing 10% (v/v) fetal bovine serum and 1% (v/v) penicillin/streptomycin. Cells (20,000/well; 200 µL) were seeded into a 96-well plate (polystyrene F-bottom, Greiner Bio-One GmbH) and incubated for 24 h (37°C, 5% CO_2_). Cells were washed with PBS (100 µL) and peptide solutions (0.6 g/L in DMEM/F-12) were added. The positive control consisted of a dilution series from 12 to 1.5% (v/v) DMSO and the negative control was 12% (v/v) PBS. After incubation (37°C, 24 h, 5% CO_2_) the supernatant was discarded, fresh medium (90 µL/well) and MTT (10 µL/well, 5 g/L in PBS) were added and the plate was incubated for 4 h (37°C, 5% CO_2_). A solution (100 µL) of sodium lauryl sulfate [10% (v/v)] in hydrochloric acid (10 mmol/L) was added and the plate was incubated again for 24 h (37°C, 5% CO_2_). The absorbance was recorded at 570 nm relative to the reference at 650 nm (PARADIGM™ microplate reader). All samples were corrected for background extinction of the medium. The relative cell viability was calculated using the ratio of the absorbance between treated and untreated cells.

## Results

### Design of ARV-1502 Derivatives and Protein Expression

**FIGURE 1:**
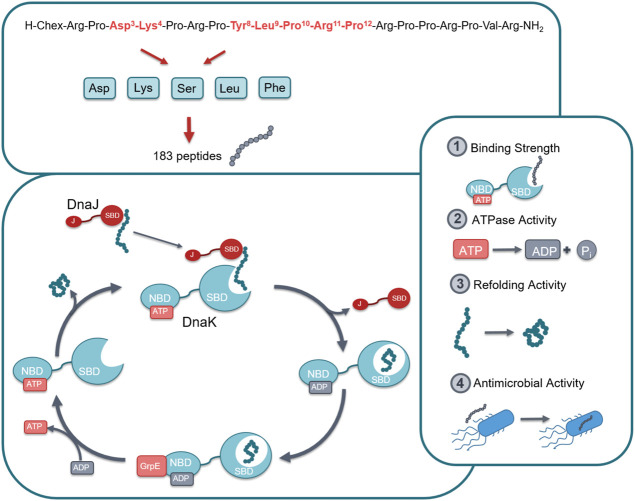
Graphical representation of the studied chaperone system. In total 183 peptides were designed and synthesized by substituting seven different positions in lead structure ARV-1502 (marked in red) by Asp, Lys, Ser, Leu or Phe in different combinations (top). All peptides were studied for their effect on the DnaK/DnaJ/GrpE-chaperone systems of *E. coli* and *S. aureus*, i.e., target binding, ATPase activity, and protein refolding activity as well as the antibacterial activity against one strain of each bacterium. Abbreviations; Chex, 1-Amino cyclohexyl carboxylic acid; SBD, Substrate binding domain; NBD, Nucleotide binding domain; J, J domain; ATP, Adenosine triphosphate; ADP, Adenosine diphosphate.

ARV-1502 binds with sequence motif YLPRP in a forward binding mode to the conventional substrate binding cleft of DnaK occupying the hydrophobic pocket (Zahn et al., 2013). These are also the residues in pyrrhocoricin that cannot be substituted without a loss in antimicrobial activity against Enterobacteriaceae. Previous investigations indicated the importance of Asp^3^ and Lys^4^ for inhibiting the ATPase activity of DnaK (Cassone et al., 2008). These are also residues that seem to be important for the anti-Gram-negative activity of pyrrhocoricin. This project was the first part of a larger research program in which a library was constructed from the residues found responsible for DnaK binding and *in vitro* activity against *E. coli*. In the first phase we had to limit ourselves to approximately 400 residues that could be tested simultaneously with high confidence. All seven residues involved, residues Asp^3^, Lys^4^, Tyr^8^, Leu^9^, Pro^10^, Arg^11^, and Pro^12^ were singly or multiply substituted with Asp (representing negatively charged side-chains), Lys (representing positively charged side-chains), Ser (representing uncharged hydrophilic side-chains), Leu (representing aliphatic side chains or no side chain functionalities at all), and Phe (representing cyclic side chains or the cyclic proline backbone). The idea was to expand the chemical space to properties not present in the original ARV-1502 monomer sequence. Thus, to reduce the number of peptides, the analogs did not include residues with “like” or chemically not different side-chains, such as Phe for Tyr or Phe and Leu for Pro. In total, the different combinations lead to 183 peptide sequences (ARV-1502 and 182 analogs) synthesized on solid phase (Supplementary Table S1). DnaK, DnaJ, and GrpE from *E. coli* and *S. aureus* were expressed in *E. coli* (DE3) Rosetta pLysS and purified via the N-terminal His-tag that was cleaved off afterwards. All proteins were obtained in high quantities and good purities (Supplementary Figure S1). The recombinant proteins were properly folded, as confirmed by the data obtained in the functional assays reported below. Thus, all peptides could be studied for their binding to *E. coli* and *S. aureus* chaperone DnaK, their impact on the ATPase activity and the protein refolding activity of DnaK in the absence or presence of co-chaperones DnaJ and GrpE, and their antibacterial activity against *E. coli* and *S. aureus* strains (Figure 1).

### DnaK Binding

**FIGURE 2:**
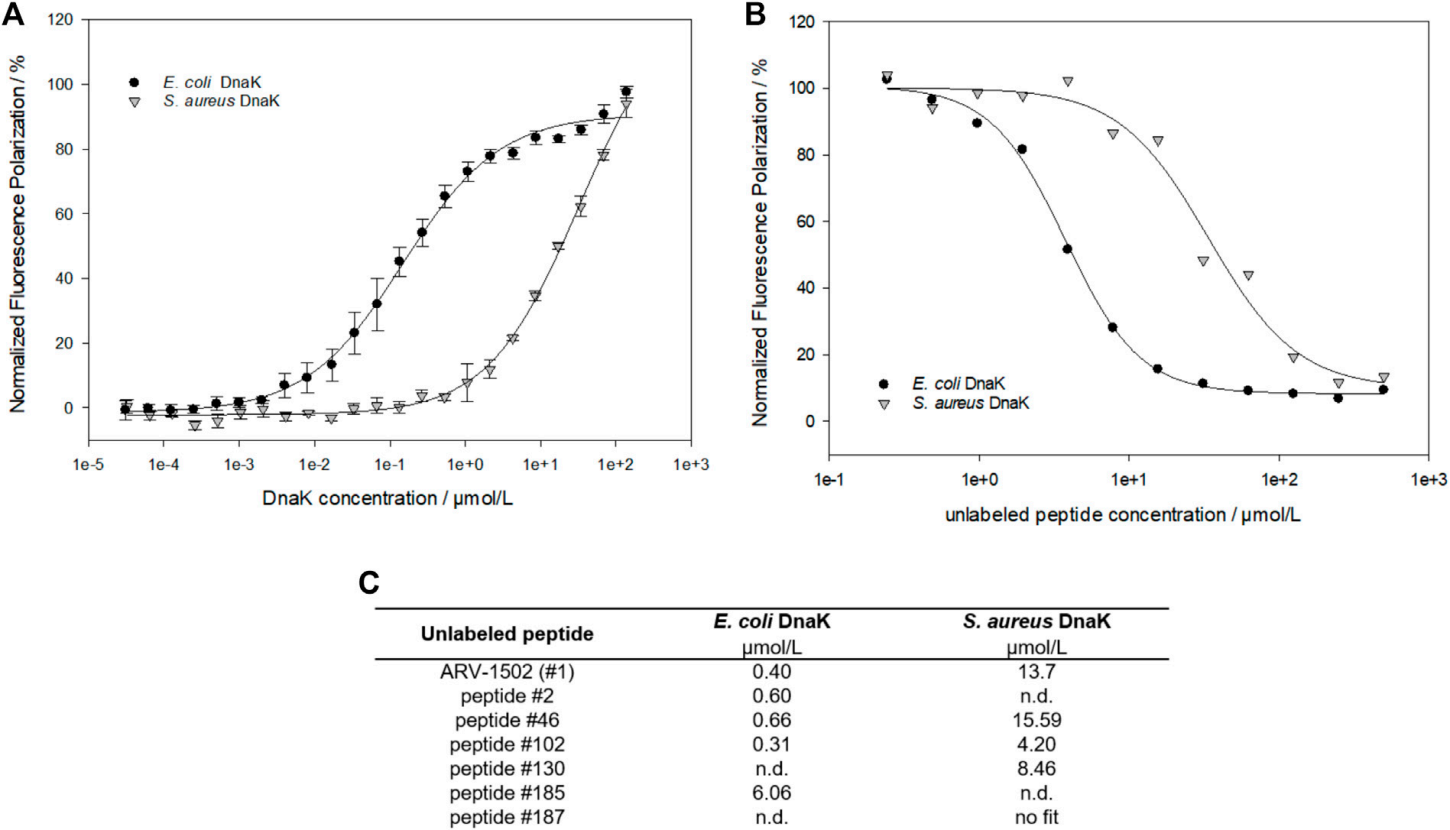
Fluorescence polarization measurements to study the interaction between ARV-1502 or substituted analogs and *E. coli* or *S. aureus* DnaK. Based on the dissociation constants (K_d_) determined for Cf-ARV-1502 **(A)**, the inhibitory constant (K_i_) was measured for ARV-1502 **(B)** and six randomly chosen screening peptides **(C)** confirming a suitable dynamic range for the screening assay.

A fluorescence polarization (FP) assay using 5 (6)-carboxyfluorescein (Cf)-labeled peptides was applied to study the binding of screening peptides to *E. coli* and *S. aureus* DnaK (Figure 2). The dissociation constants (K_d_) for Cf-ARV-1502 were 0.2 µmol/L for *E. coli* DnaK and 25.1 µmol/L for *S. aureus*, although the sequences of both DnaK variants share ∼56% identity and ∼72% similarity. The homology is even higher in the substrate binding domains, being ∼72% identity and ∼82% similarity. While the K_d_ value to *S. aureus* DnaK was not influenced by the presence of co-chaperones DnaJ and GrpE, the K_d_ value of *E. coli* DnaK increased 15-fold to 3.1 µmol/L (Table 1).

**TABLE 1:** Dissociation constants (K_d_) measured for ARV-1502 and the analog **102**. K_d_ were determined with Cf-labeled peptides and recombinant proteins DnaK, DnaJ and GrpE as *E. coli* and S. *aureus* variant. For the measurement with full chaperone system DnaK, DnaJ and GrpE (ratio 0.6:1.0:0.9) were pre-incubated for 30 min at room temperature. Fluorescence polarization of samples marked with * did not reach the upper plateau. Thus, the K_d_ is only approximate, albeit, gives a good hint of binding strength.

Peptide	K_d_/µmol/L							
	*E. coli*				*S. aureus*			
	DnaK	DnaJ	GrpE	DnaK/DnaJ/GrpE	DnaK	DnaJ	GrpE	DnaK/DnaJ/GrpE
Cf-ARV-1502	0.2	33.4*	212.8*	3.1	25.12	no fit	138.5*	30.2
Cf-peptide **102**	0.03	8.8	21.4*	0.6	1.6	53.2*	118.0*	1.6

**FIGURE 3:**
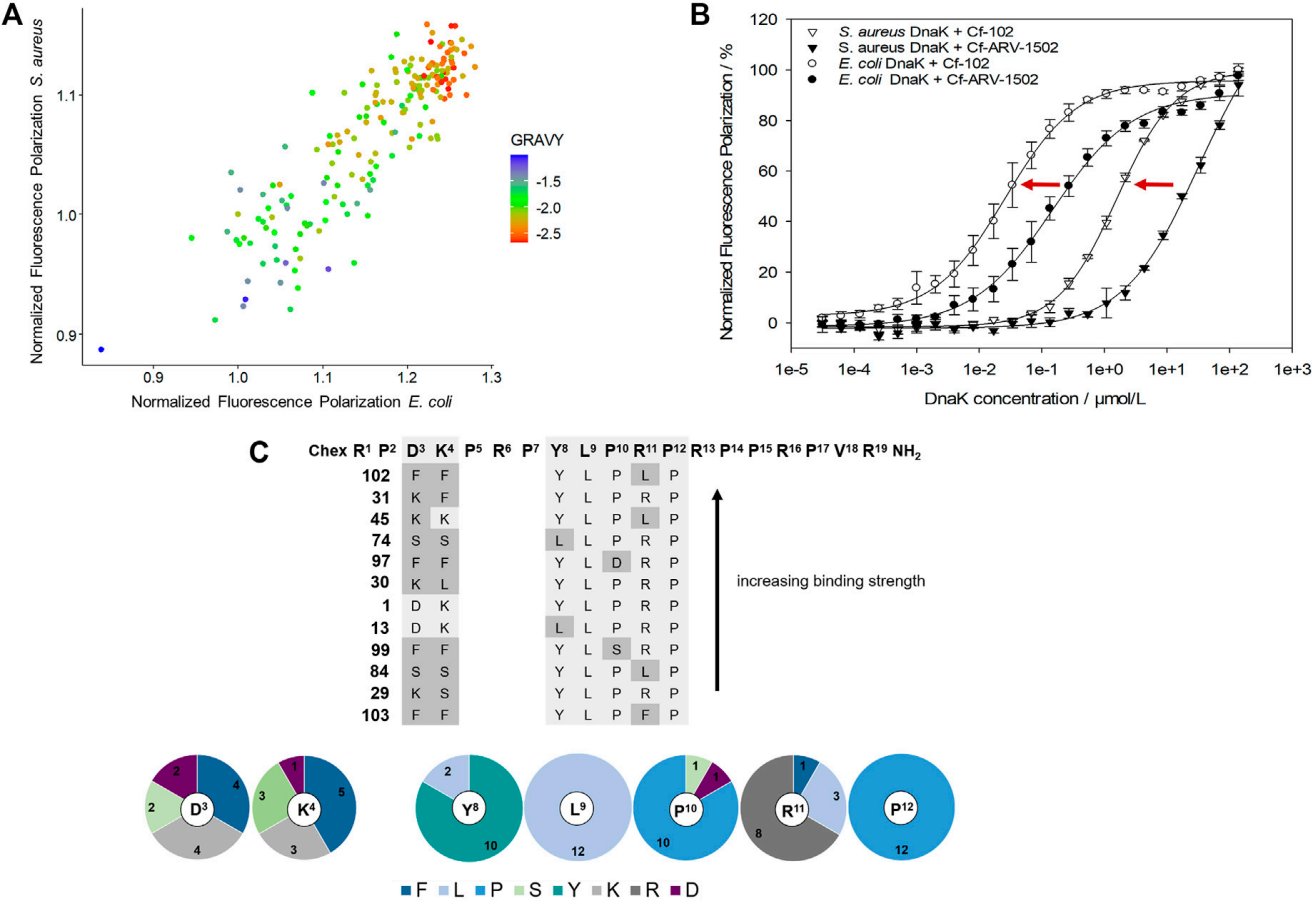
Effect of amino acid substitutions on the binding of ARV-1502 analogs to *E. coli* and *S. aureus* DnaK using fluorescence polarization (FP) values normalized to ARV-1502. Comparison of the normalized FP values for *E. coli* and *S. aureus* DnaK with the hydrophobicity of each peptide represented by the GRAVY index score indicated by the spot color, i.e., blue indicating a high hydrophobicity and red a low hydrophobicity **(A)**. The FP curves of Cf-labeled peptide **102** as the most promising screening peptide indicated a much better binding than the original ARV-1502 sequence for both DnaK variants **(B)**. Twelve peptides binding better or equal to *E. coli* and *S. aureus* DnaK than ARV-1502 (normalized FP ≤ 1) shared common features in the substitution patterns and the enrichment of certain amino acids in distinct positions [pie diagram; **(C)**].

As the determination of K_d_-values for all screening peptides would require high protein quantities and 183 Cf-labeled peptides, a competitive assay was established using Cf-ARV-1502 and unlabeled screening peptides. The DnaK concentrations were adjusted to 5 µmol/L for *E. coli* and 16 µmol/L for *S. aureus*, which was above the K_d_ at approximately 80% of the maximal plateau level. The assay was first established by determining the inhibitory constants (K_i_) of randomly chosen peptides (Figure 2 (c)), which confirmed the trend of weaker binding to *S. aureus* DnaK and provided different K_i_-values among the substituted peptides. Based on these K_i_-values, a high-throughput screening was established using the above-mentioned concentrations of DnaK and unlabeled peptide concentrations of 5 µmol/L and 35 µmol/L for *E. coli* and *S. aureus* DnaK, respectively. The quality of the experimental set up was evaluated by the Z′ factor (Zhang et al., 1999) (Supplementary Table S2). The Z′ values were 0.78 or higher indicating a reproducible measure with a large separation band between strongly and weakly binding controls. The obtained FP-values were normalized to the FP-values determined for lead sequence ARV-1502. Despite differences in the K_i_-values, substitutions showed similar effects on the binding to *E. coli* and *S. aureus* DnaK with a linear relationship (Figure 3A). Furthermore, the peptide hydrophobicity calculated as GRAVY index score correlated well to the binding strength (Figure 3A). Most hydrophilic peptides, e.g., peptides **108** (Chex-RP**DK**PRP **DKPRP**RPPRPVR-NH_2_) or **122** (Chex-RP**DK**PRP **KKPRP**RPPRPVR-NH_2_), bound neither to *E. coli* nor to *S. aureus* DnaK (Figure 3A, red dots), while the second most hydrophobic peptide **102** (Chex-RP **FF** PRP**YLPLP**RPPRPVR-NH_2_) emerged as a clear leader with 10- and 20-fold lower K_d_-values for *E. coli* (30 nmol/L) and *S. aureus* DnaK (1.6 µmol/L), respectively, than for lead structure ARV-1502 (Figure 3B, Table 1). Altogether, 42 substituted peptides bound equal or better than ARV-1502 to *S. aureus* DnaK and 12 analogs equal or better to *E. coli* DnaK, as indicated by normalized FP-values below 1 (Figure 3C). In addition to the elevated hydrophobicity, the substituted position was also important for binding, as eight of the ten best binding peptides were modified at residue 3 or 4 (mostly Phe or Lys; e.g. **31**, **45**, or **97**) and the other two peptides at residue eight or 10 (e.g. **13** or **99**), while substitutions at residues 9 and 12 weakened the interactions.

Based on the screening data obtained for DnaK, the binding of Cf-labeled peptides **102** and ARV-1502 to co-chaperones DnaJ and GrpE was also studied (Table 1). For *E. coli*, Cf-ARV-1502 and Cf-**102** bound ∼1,000- (150-) and 700- (300-) fold weaker, respectively, to GrpE (DnaJ) than to DnaK with Cf-**102** always binding stronger than Cf-ARV 1502. The differences in binding were less noticeable for the *S. aureus* proteins, as both peptides bound around 100-times weaker to *S. aureus* DnaK than to *E. coli* DnaK but showed similar K_d_-values for the co-chaperones of both bacteria (Table 1). Interestingly, the K_d_ of Cf-ARV-1502 was lower for GrpE than for DnaJ, while the K_d_ of Cf-**102** was lower for DnaJ. As observed for ARV-1502, the K_d_ of Cf-**102** was 20-fold higher for the *E. coli* DnaJ/DnaJ/GrpE-system than for DnaK alone, while the K_d_ was not affected by the *S. aureus* co-chaperones.

### ATPase Activity

The impact of ARV-1502 and its analogs on the ATPase activity of DnaK was investigated in 384-well plates using the malachite green absorbance assay, which quantitates phosphate (P_i_) cleaved from ATP confirming for example the ATPase activity of DnaK. The established assay allowed a robust and reproducible quantitation of phosphate from 10 to 150 µmol/L and was easy to handle (Supplementary Figure S3) providing the intended high-throughput screening. The ATPase activity determined here for *E. coli* DnaK corresponded very similar to the reported activities, while the ATPase activity of *S. aureus* DnaK, which has not been reported so far to the best of our knowledge, was higher (Supplementary Figure S4).

*E. coli* and *S. aureus* DnaK incubated with ARV-1502 released more P_i_ from ATP indicating that the intrinsic ATPase activity was stimulated in both cases (Supplementary Figure S5). For *E. coli* DnaK the ATPase activity increased by ∼250% reaching the plateau at ARV-1502 concentrations of 50 µmol/L or higher. The *S. aureus* DnaK was stimulated by only 50% and reached the plateau already at 25 µmol/L ARV-1502. To simulate the cellular conditions more closely, co-chaperones DnaJ and GrpE were added to the assay. Interestingly, the ATPase activity of the *E. coli* DnaK/DnaJ/GrpE-chaperone system was downregulated in the presence of ARV-1502 by ∼70%, whereas a stimulatory effect of ∼50% was observed for the *S. aureus* system, i.e., at a similar degree as for DnaK alone (Supplementary Figure S5).

**FIGURE 4:**
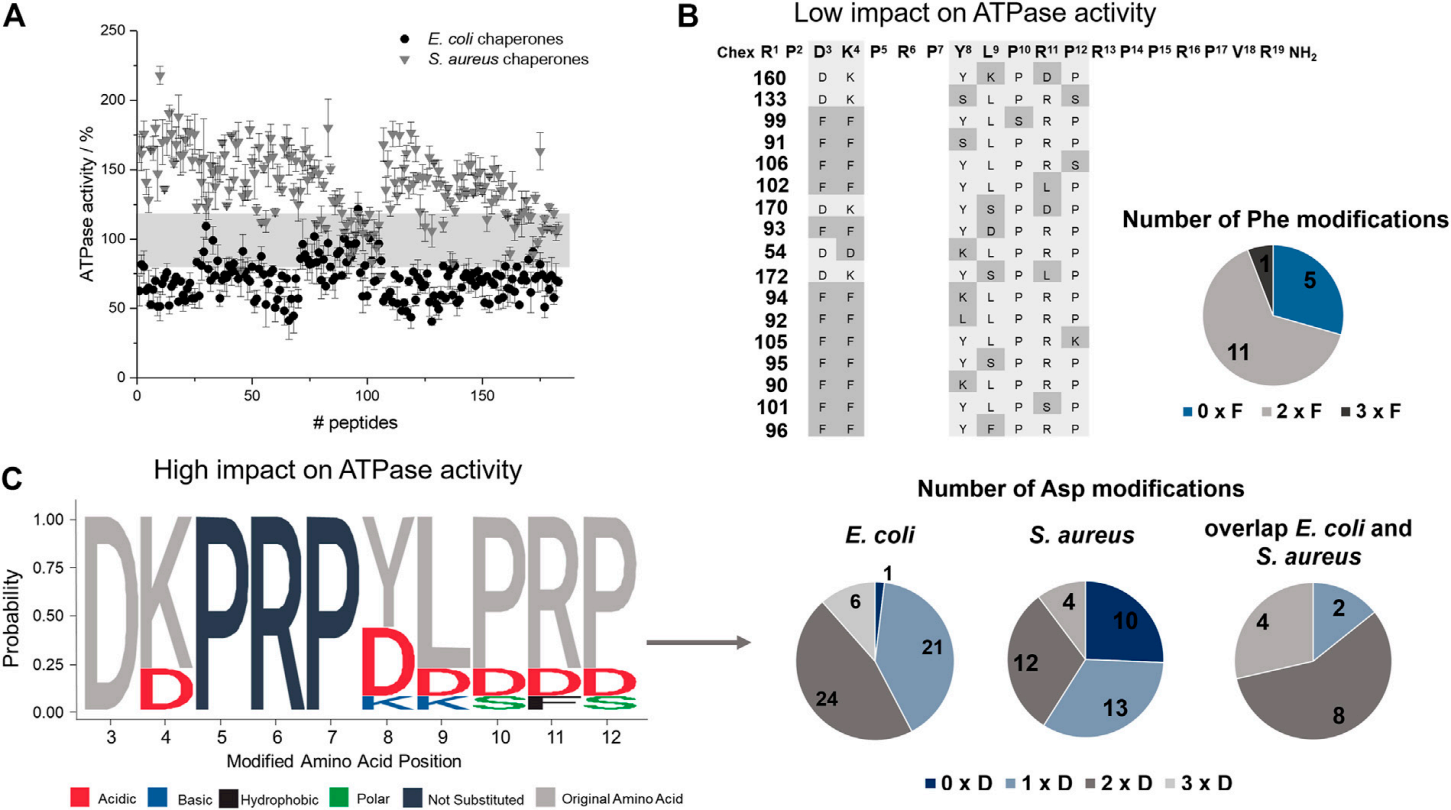
Effect of ARV-1502 or its substituted analogs on the ATPase activity of the DnaK/DnaJ/GrpE chaperone system. The phosphate released from ATP at 37°C was determined after 2 hours using a malachite-green absorbance assay. The ATPase activity determined for the *E. coli* (●) and *S. aureus* (▼) chaperone system in the presence of a peptide was normalized to the activity recorded in the absence of a peptide (100%). The range from 80 to 120% ATPase activity is highlighted in grey **(A)**. The sequences of 17 peptides in this activity range of ±20% were enriched for Phe-substitutions, which are marked in grey, with typically two Phe substitutions at the same time **(B)**. Logo plot of sequences with strong inhibitory or stimulating effects on the ATPase activity of *E. coli* (activity <60%) and *S. aureus* (activity >160%), respectively. The logo plot was created with the R package (Wagih, 2017). Dark grey letters indicate unmodified positions and light grey letters the common presence of the original amino acid, while the color code indicates the property of the substitution. The pie diagrams indicate the number of the sequences with no, one, two or three Asp modifications **(C)**.

The peptide screen relied on high peptide concentrations (0.3 mmol/L) to reduce the effect of different adsorption rates of hydrophobic peptides on surfaces and to observe a strong effect on the ATPase activity of the DnaK/DnaJ/GrpE-chaperone system, mostly at the plateau level. The ATPase activity of the *E. coli* system ranged from 41 to 122% with 52 peptides (28%; e.g., **66**, **117**, or **128**) decreasing the activities below 60% and of the *S. aureus* system from 73 to 218% with 39 peptides (21%; e.g., **10**, **83**, **119**) increasing the activities by more than 160% (Figure 4). Surprisingly, 17 peptides affected the ATPase activities of *E. coli* and *S. aureus* chaperones by less than 20% (e.g., **99**, **106**, or **160**), i.e., observed activities between 80 and 120%. Strikingly, seven of these 17 peptides (41%; **90**, **91**, **99**, **101**, **102**, **105**, and **106**) belonged to best binders for *E. coli* and *S. aureus* DnaK and additional five peptides (**54**, **133**, **160**, **170**, and **172**) to the best binders for *S. aureus* DnaK. The Phe^3^-Phe^4^ motif appeared to be very important, as it was present in twelve of the 17 peptides (e.g., **91**, **99**, **102**). Peptides showing the strongest effects of the ATPase activities of *E. coli* (<60%) or *S. aureus* (>160%) contained typically the original Asp^3^ or Lys^4^, although Asp was also frequently present at position 4. In general, Asp substitutions occurred quite often in these sequences, i.e., 58% of the peptides decreasing the ATPase activity of *E. coli* DnaK by at least 40% and contained at least two Asp residues (e.g., **66**, **119**, or **128**), while 41% of the peptides increasing the ATPase activity of *S. aureus* DnaK by 60% or more had at least two Asp spread on the substituted positions (e.g., **53**, **107**, or **117**). Interestingly, none of these peptides bound to DnaK.

### Refolding Activity

**FIGURE 5:**
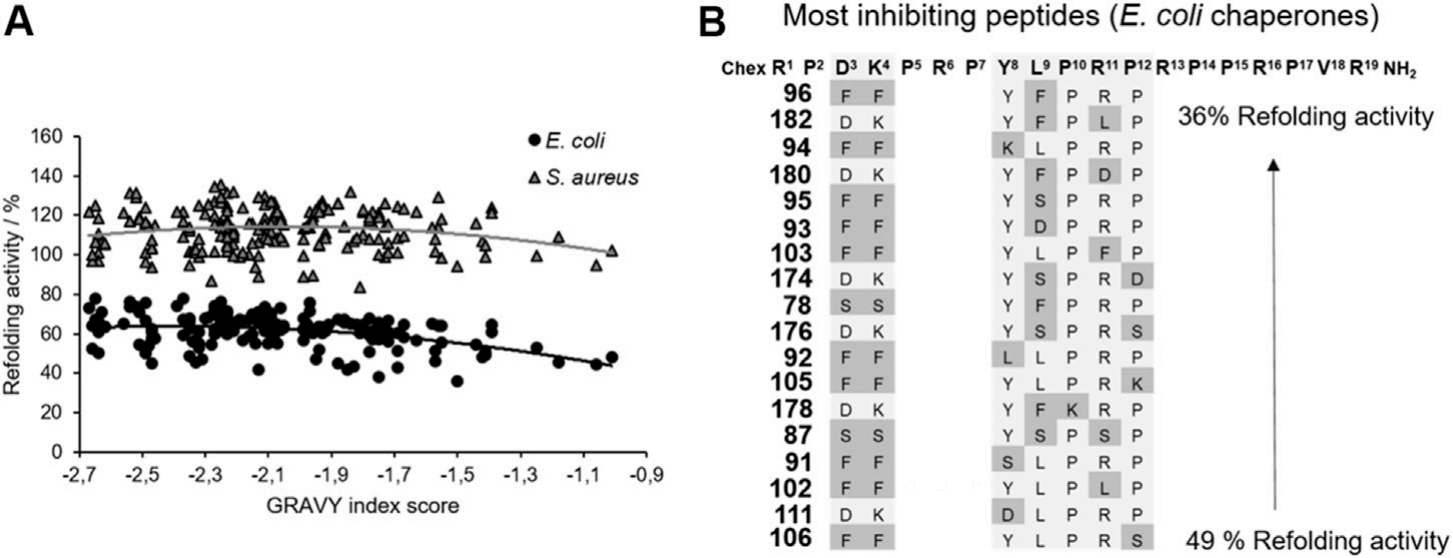
Impact of ARV-1502 or its substituted analogs on the refolding activity of the *E. coli* and *S. aureus* chaperone systems using a denatured luciferase refolding assay. Refolding activity of the *E. coli* (●) and *S. aureus* (▲) chaperone systems in the presence of a substituted ARV-1502 analog relative to the chaperone system in the absence of a peptide (100%). The peptides were sorted by their hydrophobicity based on the GRAVY index score **(A)**. Sequence alignment of 18 peptides inhibiting the refolding activity of the *E. coli* chaperone systems by at least 50%. Substituted positions are marked in darker grey **(B)**.

The main function of the DnaK chaperone system is the refolding and degradation of denatured or misfolded proteins enabling cell survival after severe stress conditions (Mayer and Bukau, 2005), such as a heat shock. The effect of ARV-1502 analogs on the refolding activity of the DnaK chaperone system was studied in a denatured luciferase refolding assay. When firefly luciferase was denatured with guanidinium hydrochloride, the luminescence intensity of the luciferase assay decreased to 5%. After dilution allowing the protein refolding, the luminescence intensity increased in the absence of a chaperone to only 10% after 2 hours (Supplementary Figure S6). ARV-1502 reduced the refolding activity of the *E. coli* DnaK/DnaJ/GrpE-chaperone system to ∼60% at peptide concentrations ≥50 µmol/L, while the *S. aureus* chaperone system was not affected (Supplementary Figure S7). The peptide screening led to similar results, i.e., an inhibition of the *E. coli* chaperone refolding activity by 22–64%, whereas the *S. aureus* chaperone system was slightly inhibited or slightly activated with activities ranging from 84 to 135%. Although the impact was different, similar trends were observed for the substituted peptides indicating that inhibition of both chaperone systems increased with higher peptide hydrophobicity (GRAVY index; Figure 5). The Phe^3^-Phe^4^-motif, which improved DnaK-binding without affecting the ATPase activity, was present in 10 of the 20 peptides (e.g., **96**, **102**, or **105**) most strongly inhibiting the *E. coli* chaperone system. Additionally, hydrophobic amino acids phenylalanine and leucine were favored at position 9, i.e., in 60% of the peptides (e.g., **94**, **111**, **182**). A substitution of Tyr^8^ did neither increase nor reduce the inhibitory effect. Generally, the overall peptide hydrophobicity appeared to be more important than the substituted position for the refolding activity.

### Antimicrobial Activity and Cytotoxicity

The MIC-values obtained for the Gram-negative strain *E. coli* BW25113 ranged from 4 µg/mL to >128 µg/mL with only peptide **2** being more active than the lead structure ARV-1502 with an MIC of 8 µg/mL determined in parallel. ARV-1502 was inactive against the Gram-positive strain *S. aureus* DSM 6247, but this strain was susceptible to several substituted peptides with the lowest MIC-values at 16 µg/mL. In total 68 and 76% of the tested peptides were inactive (MIC ≥128 µg/mL) against *E. coli* and *S. aureus*, respectively, which might relate to changes in the physicochemical properties as a result of some substitutions.

**FIGURE 6:**
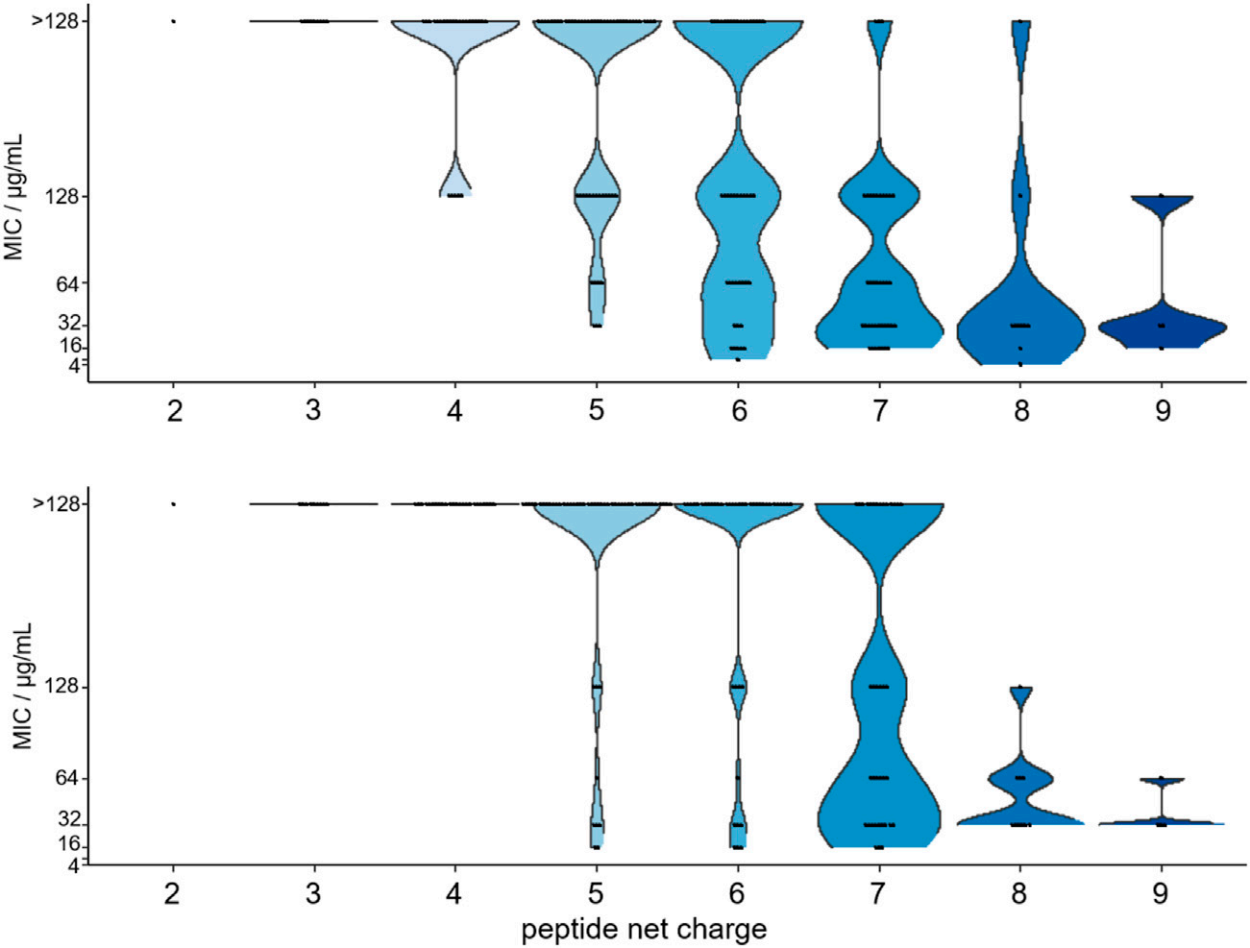
Violin plot of the MIC-values determined for 179 substituted ARV-1501 peptides against *E. coli* (above) and *S. aureus* (below) versus the peptide net charge expressed as difference of basic and acidic residues in each peptide sequence.

Importantly, the antimicrobial activity was affected by the net charge. Inactive peptides (MIC ≥128 µg/mL) had net charges below +5, whereas net charges above +7 resulted mostly in MICs of 32 µg/mL or lower (Figure 6). Particularly, substitutions with Asp strongly decreased the antimicrobial activity. All six sequences with a single Asp-substitution (Table 2 (a)) as well as 89 and 94% of the peptides, where at least one Asp-substitution was combined with other substitutions, lost the antimicrobial activity against *E. coli* and *S. aureus*, respectively. Reversing the motif Asp^3^-Lys^4^ to Lys^3^-Asp^4^ reduced the activity only two-fold to 16 µg/mL for *E. coli* and had no effect for *S. aureus*. Similar to Asp-substitutions, Ser-substitutions at positions Tyr^8^, Pro^10^, Arg^11^, and Pro^12^ as well as for 70% or 80% of all peptides carrying multiple substitutions involving serine abolished the antimicrobial activity against *E. coli* or *S. aureus* (Table 2 (a)). Positions Tyr^8^ and Leu^9^ appeared to be most critical, as an exchange of these residues reduced the activity against *E. coli* and *S. aureus* significantly for 71 and 80% of these peptides (e.g., **10**, **54**, or **64**), respectively. Less negative effects were observed for substituting the Asp^3^-Lys^4^ motif in ARV-1502 with Lys or Phe to obtain the Lys^3^-Lys^4^ or Phe^3^-Phe^4^ motifs exhibiting good activities. All peptides tested against *S. aureus* with MIC-values ≤ 16 µg/mL contained the Phe^3^-Phe^4^-motif (**92**, **94**, **98**, **99**, **102**, **103**, and **105**). Altogether, only peptide **2** of all designed peptides was more active against the Gram-negative *E. coli* strain. Particularly noteworthy is that 32 peptides improved the activity against *S. aureus* by at least three dilution steps (MIC ≤32 µg/mL). When the antimicrobial activity of peptides **1** (ARV-1502), **10**, **45**, and **102** was tested against the *∆dnaK*-knock-out-mutant *E. coli* JW0013 (Table 2 (b)), the MIC-values increased or decreased only twofold. The most active peptides (≤16 µg/mL) were analyzed for possible cytotoxic effects on eukaryotic cells (HEK293 and HepG2) using an MTT-based colorimetric assay and peptide concentrations of 0.6 g/L (Supplementary Figure S8). For 86% of the tested peptides, a lower viability was observed for HepG2. In addition, hydrophobic peptides, such as peptide **105** (Chex-RPFFPRPYLPRKRPPRPVR-NH_2_) and **103** (Chex-RPFFPRPYLPFPRPPRPVR-NH_2_), exhibited a stronger cytotoxic effect, resulting in a minimum viability of 39% (±5%) in HepG2 and 61% (±6%) in HEK293 cells. However, on average, a relative viability of 78% (±4%) was achieved for HepG2 and 91% (±5%) for HEK293 cells.

**TABLE 2:** MIC values measured for *E. coli* BW25113, *S. aureus* DSM 6247 and the knock-out mutant *E. coli* JW0013 ∆dnaK in 25% MHB2. a) Sequences with a single Asp or Ser substitution showed significantly lower antimicrobial activities in *E. coli* BW25113 or *S. aureus* DSM 6247. b) Peptides with similar antibacterial activities against both strains. These peptides were chosen due to their behavior in the other screening (peptide 10: insufficient binding; 45: insufficient binding, but high impact on ATPase activity; 102: best binding).

(a)					
Sequences with a single asp substitution	MIC/µg/mL		Sequences with a single Ser substitution	MIC/µg/mL	
	*E. c.*	*S. a.*		*E. c.*	*S. a.*
Chex-RP**DK**PRP**YLPRP**RPPRPVR-NH_2_	8	>128	Chex-RP**SK**PRP**YLPRP**RPPRPVR-NH_2_	n. d.	128
Chex-RP**DD**PRP**YLPRP**RPPRPVR-NH_2_	128	>128	Chex-RP**DS**PRP**YLPRP**RPPRPVR-NH_2_	32	>128
Chex-RP**DK**PRP**DLPRP**RPPRPVR-NH_2_	>128	>128	Chex-RP**DK**PRP**SLPRP**RPPRPVR-NH_2_	128	>128
Chex-RP**DK**PRP**YDPRP**RPPRPVR-NH_2_	>128	>128	Chex-RP**DK**PRP**YSPRP**RPPRPVR-NH_2_	64	>128
Chex-RP**DK**PRP**YLDRP**RPPRPVR-NH_2_	>128	>128	Chex-RP**DK**PRP**YLSRP**RPPRPVR-NH_2_	128	>128
Chex-RP**DK**PRP**YLPDP**RPPRPVR-NH_2_	>128	>128	Chex-RP**DK**PRP**YLPSP**RPPRPVR-NH_2_	>128	>128
Chex-RP**DK**PRP**YLPRD**RPPRPVR-NH_2_	>128	>128	Chex-RP**DK**PRP**YLPRS**RPPRPVR-NH_2_	>128	>128

(b)			
		MIC/µg/mL	
#	Peptide Sequence	*E. coli* BW25113	*E. coli* JW0013 ∆dnaK
1	Chex-RP**DK**PRP**YLPRP**RPPRPVR-NH_2_	8	4-8
10	Chex-RP**DK**PRP**YDPRP**RPPRPVR-NH_2_	32-64	32-64
45	Chex-RP**KK**PRP**YLPRL**RPPRPVR-NH_2_	>128	>128
102	Chex-RP**FF**PRP**YLPLP**RPPRPVR-NH_2_	16	32

## Discussion

**FIGURE 7:**
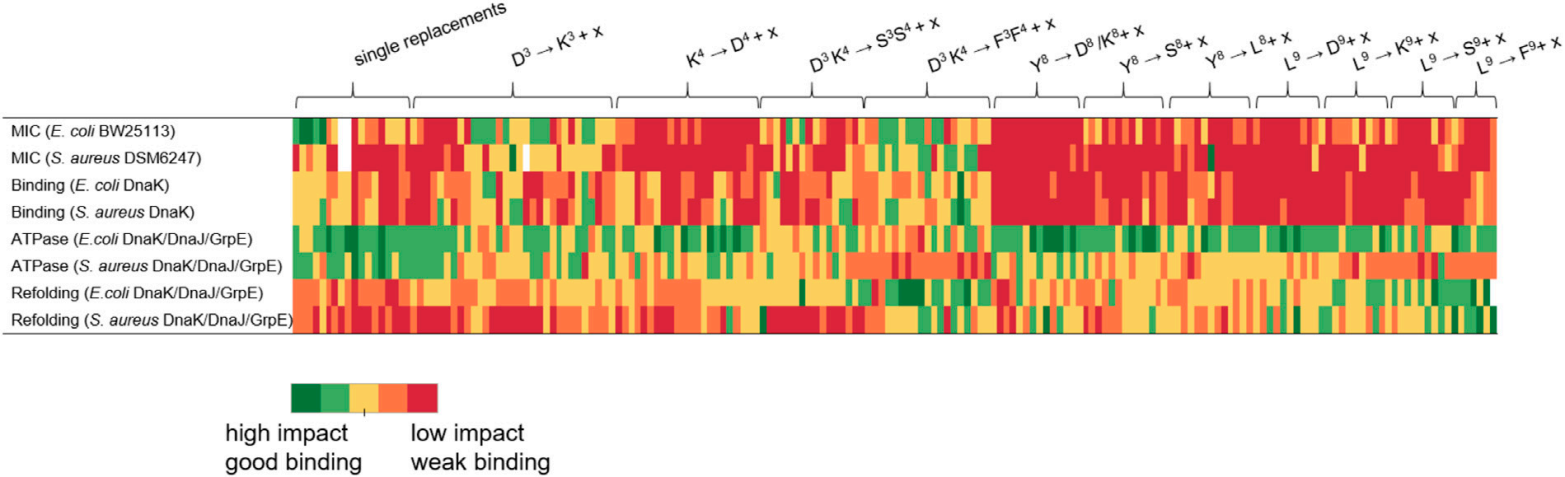
Graphical representation of the influence strength of the studied peptides grouped by substituted amino acids. x represents one of the five substituted amino acids Asp, Lys, Ser, Leu or Phe. Peptides with high impact or good DnaK binding are shown in green and those with low efficacy or poor binding are shown in red. For the assignment of the color scale, the results of the respective tests were divided into five equal groups corresponding to the five color groups used.

Proteins are involved in almost all biological processes, which demands their correct folding. This is a very complex process requiring cellular chaperone systems. Increased or insufficient activity of chaperones can lead to misfolded proteins, which are often functionally inactive, or trigger protein aggregation. Human Hsp70 is associated with cancers or neurodegenerative diseases, including Alzheimer’s and Parkinson’s disease (Wang et al., 2014). Such effects can be exploited *in vitro*, typically using bacterial homologs, often referred to as DnaK, as promising targets. Additionally, DnaK inhibitors might represent a new class of antimicrobial agents. This study investigated the effect of PrAMP ARV-1502 and 182 substituted analogs on different DnaK functions, specifically binding strength, ATPase activity, and refolding activity, also in association with co-chaperones DnaJ and GrpE. In addition to the well-established *E. coli* model system, the corresponding *S. aureus* system was included as a model of Gram-positive bacteria. Despite the high sequence homologies between the chaperones and co-chaperones of both bacteria, the binding domains are different, which may influence their interactions and thus require different inhibitors. All studied peptides were 19 residues long and differed only by a maximum of three substitutions at seven different positions. Nevertheless, major differences and trends were observed in all functional assay types. Substitution with Asp, Lys, Ser, Leu or Phe changed the peptide properties, mainly by the hydrophobicity and net charge, resulting in varying degrees of interference between the different assay types (Figure 7). Not surprisingly, inhibition of DnaK activity correlated well with the antibacterial activity.

DnaK prefers substrate peptides enriched for aliphatic (Leu and Ile) or aromatic amino acids (Phe and Tyr) with leucine being the most important (Rüdiger et al., 1997). Leucine is also essential for the binding of ARV-1502, which binds in a forward binding mode to the central hydrophobic pocket of the substrate binding domain (SBD) (Zahn et al., 2013). The importance of Leu^9^ was confirmed here, as none of the twelve peptides binding best to DnaK was substituted in position 9. However, some peptides contained additional leucines at other positions (e.g., peptides **23**, **45**, or **102**). In general, only few modifications at the binding motif Y^8^LPRP^12^ increased the binding strength for both DnaK variants, not even a substitution with the hydrophobic phenylalanine. A likely reason is the limited space in the central pocket, where bulky residues can bind only after reorientation (Mayer et al., 2000; Mayer and Bukau, 2005). Interestingly, the peptide binding best (peptide **102**) contained a Phe^3^-Phe^4^ motif instead of Asp^3^-Lys^4^. This drastic change could possibly lead to a different binding mode enhancing the binding through additional interactions with the SBD. It is particularly noteworthy that the K_d_-values determined for *S. aureus* DnaK were much higher than for *E. coli*, although both variants and their SBD share high homologies. Small changes of central hydrophobic pocket, such as the exchange of Phe^426^ to Tyr, might explain the difference. In addition, the SBD of *S. aureus* DnaK has a lower isoelectric point than the corresponding *E. coli* sequence influencing electrostatic interactions (Rüdiger et al., 1997). Stronger DnaK binding of the primarily hydrophobic peptides increased also the inhibition of the DnaK-refolding activity (e.g., peptides **92**, **102**, or **103**). When the SBD is occupied by a peptide, it cannot bind to denatured luciferase and thus cannot promote its refolding to the native state. ARV-1502 added at concentrations of ≥50 µmol/L reduced the refolding activity of the *E. coli* system to only 60%, while substituted analogs with hydrophobic character reduced it even further to 36% (e.g., peptides **78**, **94**, **96**, or **105**), always reaching the basal refolding level of 10% in the absence of a chaperone. The relevance of hydrophobicity for the binding mode is known from studies on small molecules, e.g., dihydropyrimidines, with EC_50_-values in the lower micromolar range (Wisén and Gestwicki, 2008; Liebscher et al., 2010). The inhibition reached seems to be limited by the peptide size. However, it should be noted that the inhibitory and stimulatory effects did not only depend on the binding to DnaK but were also influenced by co-chaperones DnaJ and GrpE, important for the transfer and release of DnaK substrates. This was also reported for other inhibitors or activators (Cesa et al., 2013), which are able to induce conformational changes or suppress the chaperone-co-chaperone-interaction (Liebscher et al., 2010). For the *E. coli* chaperone system, the addition of co-chaperones reversed the effect of ARV-1502 from stimulation to inhibition of the ATPase activity of DnaK along with 15-fold higher K_d_-values. Interestingly, the best binding peptides (e.g., peptides **102**, **103**, and **106**) showed the weakest effects on the ATPase activity, whereas peptides that did not bind to DnaK, decreased the ATPase activity by 50% (e.g., peptides **68**, **119**, **128**). Since ARV-1502 bound more weakly to *E. coli* DnaK, DnaJ, and GrpE than peptide **102**, but affected the ATPase activity more strongly, we assume that the binding strength is not the leading parameter for designing inhibitors. The influence of the co-chaperones on the peptide-DnaK binding strength probably affected also the impact on the ATPase and refolding activities. Notably, weakly binding peptides inhibited the ATPase activity of *E. coli* chaperones, but stimulated the corresponding *S. aureus* chaperones. Again, strongly binding peptides did not affect the ATPase activity in either system (e.g., peptides **91**, **99**, or **102**). Considering the antimicrobial activity, only the Asp^3^Lys^4^ substitution slightly improved the MIC-value for *E. coli*, while substitutions of the original ARV-1502 sequence in the binding motif YLPRP always reduced the activity (peptides **10** to **27** and **107**–**183**), although peptides **158** (YKKRP) and **178** (YFKRP) were more active against *S. aureus* (MIC = 32 µg/mL). In general, substitutions with Asp and Ser were unfavorable, most likely due to poor interactions with the cell wall and the outer cell membrane (Welch et al., 2020). In contrast, the antimicrobial activity against *S. aureus* was significantly improved. This is very encouraging, especially for the Phe^3^-Phe^4^-motif present in most highly active sequences (e.g., peptides **90**, **92**, **94**, **102**, and **106**). Again, it can be assumed that the cellular uptake is improved and that slight lytic effects may also occur (Matsuzaki, 2009; Lee et al., 2014). As shown in previous studies, the antimicrobial activity testing with the DnaK knockout mutant confirmed that DnaK is not the primary lethal target for PrAMPs, but rather can be described as a secondary non-lethal target (Krizsan et al., 2014; Knappe et al., 2016). In addition, some peptides were found to inhibit the ATPase and refolding activity of *E. coli* DnaK better than the lead sequence without improving the antimicrobial activity (e.g., peptides **66**, **111**, and **159**). Inhibition of DnaK is particularly harmful for bacteria, especially in stressful situations, leading to cell death (Paek and Walker, 1987). However, since PrAMPs, such as ARV-1502-like Onc112, bind and act at the 70S ribosome of multiple bacteria and translation goes hand in hand with protein folding, a dual mode of action and binding to two different targets could be beneficial for the antimicrobial activity and reduce the probability of target-related resistances (Kolano et al., 2020).

## Conclusion

The screening of 182 analogs derived from the lead PrAMP ARV-1502 provided interesting results allowing a better understanding of their effect on the functional activity of *E. coli* and *S. aureus* DnaK and its co-chaperones. Similarities and differences between the Gram-negative and Gram-positive chaperone systems could be identified, which could help to develop antibacterials targeting the bacterial chaperone system or to target the human Hsp70 chaperones to treat chaperone-related diseases including cancer. Substitutions with phenylalanine in ARV-1502 improved the binding to DnaK, inhibition of refolding activity, and antimicrobial activity against *S. aureus*. A stronger influence on the functionality of the chaperone system did not necessarily increase the antimicrobial activity, which confirms that inhibition of the DnaK chaperone system does not kill the bacteria, at least when using the favorable growth conditions applied for determining the MIC-values *in vitro*.

## Data Availability

The dataset presented in this study can be found in the Supplementary Material (Table S3).
