# Supplementary material for: Functional Effects of ARV-1502 Analogs Against Bacterial Hsp70 and Implications for Antimicrobial Activity
Source: Front Chem. 2022 Feb 9;10:798006. doi: 10.3389/fchem.2022.798006 (PMC8864165; doi:10.3389/fchem.2022.798006)
Supplement: Supplementary file 1 [file DataSheet1.pdf]

Supplementary information

Functional effects of ARV-1502 analogs against bacterial Hsp70 and implications for antimicrobial activity

Alexandra Brakel, Lisa Kolano, Carl N. Kraus, Laszlo Otvos, Jr and Ralf Hoffmann

Table of Contents

Table S1 Sequences of all investigated peptides........S2

Table S2 Z'-values calculated for the established high-throughput screening S4

Table S3 Summarized data of performed assays S5

Figure S1 SDS-PAGE of purified recombinant chaperone........ S10

Figure S2 Reproducibility of optimized FP-assay S10

Figure S3 Validation of ATPase activity assay........ S11

Figure S4 ATPase activity of in-house expressed chaperones........ S12

Figure S5 ATPase activity after incubation with ARV-1502 .... S12

Figure S6 Luminescence intensity obtained for controls of luciferase refolding activity assay S13

Figure S7 Refolding activity of chaperones after incubation with ARV-1502 ....S13

Figure S8 Cytotoxicity assay........S14

Table S1: Sequences of all investigated peptides and the corresponding GRAVY index scores and isoelectric points (pI) calculated with the "Peptide Analyzing Tool" (Thermo Fisher Scientific Inc, Watham, USA). The substituted positions are highlighted in bold, the substituted residue is underlined. The non-natural Chex-residue (1-Amino cyclohexyl carboxylic acid) could not be considered in the calculation, but should have a similar effect on all peptides.

#	Sequence	GRAVY	pI
1	Chex-RP**DK**PRP**YLPRP**RPPRPVR-NH$_2$	-2.13	12.5
2	Chex-RP**KK**PRP**YLPRP**RPPRPVR-NH$_2$	-2.15	12.8
3	Chex-RP**SK**PRP**YLPRP**RPPRPVR-NH$_2$	-1.99	12.8
4	Chex-RP**LK**PRP**YLPRP**RPPRPVR-NH$_2$	-1.75	12.8
5	Chex-RP**FK**PRP**YLPRP**RPPRPVR-NH$_2$	-1.8	12.8
6	Chex-RP**DD**PRP**YLPRP**RPPRPVR-NH$_2$	-2.11	12.2
7	Chex-RP**DS**PRP**YLPRP**RPPRPVR-NH$_2$	-1.97	12.5
8	Chex-RP**DL**PRP**YLPRP**RPPRPVR-NH$_2$	-1.73	12.5
9	Chex-RP**DF**PRP**YLPRP**RPPRPVR-NH$_2$	-1.78	12.5
10	Chex-RP**DK**PRP**DLPRP**RPPRPVR-NH$_2$	-2.25	12.5
11	Chex-RP**DK**PRP**KLPRP**RPPRPVR-NH$_2$	-2.27	12.8
12	Chex-RP**DK**PRP**SLPRP**RPPRPVR-NH$_2$	-2.11	12.8
13	Chex-RP**DK**PRP**LLPRP**RPPRPVR-NH$_2$	-1.86	12.8
14	Chex-RP**DK**PRP**YDPRP**RPPRPVR-NH$_2$	-2.52	12.2
15	Chex-RP**DK**PRP**YKPRP**RPPRPVR-NH$_2$	-2.54	12.5
16	Chex-RP**DK**PRP**YSPRP**RPPRPVR-NH$_2$	-2.37	12.5
17	Chex-RP**DK**PRP**YFPRP**RPPRPVR-NH$_2$	-2.18	12.5
18	Chex-RP**DK**PRP**YLDRP**RPPRPVR-NH$_2$	-2.23	12.2
19	Chex-RP**DK**PRP**YLKRP**RPPRPVR-NH$_2$	-2.25	12.5
20	Chex-RP**DK**PRP**YLSRPR**PPRPVR-NH$_2$	-2.09	12.5
21	Chex-RP**DK**PRP**YLPDP**RPPRPVR-NH$_2$	-2.08	11.9
22	Chex-RP**DK**PRP**YLPSP**RPPRPVR-NH$_2$	-1.94	12.3
23	Chex-RP**DK**PRP**YLPLP**RPPRPVR-NH$_2$	-1.69	12.3
24	Chex-RP**DK**PRP**YLPFP**RPPRPVR-NH$_2$	-1.75	12.3
25	Chex-RP**DK**PRP**YLPRD**RPPRPVR-NH$_2$	-2.23	12.2
26	Chex-RP**DK**PRP**YLPRK**RPPRPVR-NH$_2$	-2.25	12.5
27	Chex-RP**DK**PRP**YLPRS**RPPRPVR-NH$_2$	-2.09	12.5
28	Chex-RP**KD**PRP**YLPRP**RPPRPVR-NH$_2$	-2.13	12.5
29	Chex-RP**KS**PRP**YLPRP**RPPRPVR-NH$_2$	-1.99	12.8
30	Chex-RP**KL**PRP**YLPRP**RPPRPVR-NH$_2$	-1.75	12.8
31	Chex-RP**KF**PRP**YLPRP**RPPRPVR-NH$_2$	-1.8	12.8
32	Chex-RP**KK**PRP**DLPRP**RPPRPVR-NH$_2$	-2.27	12.8
33	Chex-RP**KK**PRP**KLPRP**RPPRPVR-NH$_2$	-2.29	13.2
34	Chex-RP**KK**PRP**SLPRP**RPPRPVR-NH$_2$	-2.13	13.2
35	Chex-RP**KK**PRP**LLPRP**RPPRPVR-NH$_2$	-1.88	13.2
36	Chex-RP**KK**PRP**YDPRP**RPPRPVR-NH$_2$	-2.54	12.5
37	Chex-RP**KK**PRP**YKPRP**RPPRPVR-NH$_2$	-2.56	12.8
38	Chex-RP**KK**PRP**YSPRP**RPPRPVR-NH$_2$	-2.39	12.8
39	Chex-RP**KK**PRP**YFPRP**RPPRPVR-NH$_2$	-2.21	12.8
40	Chex-RP**KK**PRP**YLDRP**RPPRPVR-NH$_2$	-2.25	12.5
41	Chex-RP**KK**PRP**YLKRP**RPPRPVR-NH$_2$	-2.27	12.8
42	Chex-RP**KK**PRP**YLSRP**RPPRPVR-NH$_2$	-2.11	12.8
43	Chex-RP**KK**PRP**YLPDP**RPPRPVR-NH$_2$	-2.1	12.3
44	Chex-RP**KK**PRP**YLPSP**RPPRPVR-NH$_2$	-1.96	12.7
45	Chex-RP**KK**PRP**YLPLP**RPPRPVR-NH$_2$	-1.72	12.7
46	Chex-RP**KK**PRP**YLPFP**RPPRPVR-NH$_2$	-1.77	12.7
47	Chex-RP**KK**PRP**YLPRD**RPPRPVR-NH$_2$	-2.25	12.5
48	Chex-RP**KK**PRP**YLPRK**RPPRPVR-NH$_2$	-2.27	12.8
49	Chex-RP**KK**PRP**YLPRS**RPPRPVR-NH$_2$	-2.11	12.8
50	Chex-RP**SD**PRP**YLPRP**RPPRPVR-NH$_2$	-1.97	12.5
51	Chex-RP**LD**PRP**YLPRP**RPPRPVR-NH$_2$	-1.73	12.5
52	Chex-RP**FD**PRP**YLPRP**RPPRPVR-NH$_2$	-1.78	12.5
53	Chex-RP**DD**PRP**DLPRP**RPPRPVR-NH$_2$	-2.23	12.2
54	Chex-RP**DD**PRP**KLPRP**RPPRPVR-NH$_2$	-2.25	12.5
55	Chex-RP**DD**PRP**SLPRP**RPPRPVR-NH$_2$	-2.08	12.5
56	Chex-RP**DD**PRP**LLPRP**RPPRPVR-NH$_2$	-1.84	12.5
57	Chex-RP**DD**PRP**YDPRP**RPPRPVR-NH$_2$	-2.49	11.8
58	Chex-RP**DD**PRP**YKPRP**RPPRPVR-NH$_2$	-2.52	12.2
59	Chex-RP**DD**PRP**YSPRP**RPPRPVR-NH$_2$	-2.35	12.2
60	Chex-RP**DD**PRP**YFPRP**RPPRPVR-NH$_2$	-2.16	12.2
61	Chex-RP**DD**PRP**YLDRP**RPPRPVR-NH$_2$	-2.21	11.8
62	Chex-RP**DD**PRP**YLKRP**RPPRPVR-NH$_2$	-2.23	12.2
63	Chex-RP**DD**PRP**YLSRP**RPPRPVR-NH$_2$	-2.07	12.2
64	Chex-RP**DD**PRP**YLPDP**RPPRPVR-NH$_2$	-2.06	11
65	Chex-RP**DD**PRP**YLPSP**RPPRPVR-NH$_2$	-1.92	11.9
66	Chex-RP**DD**PRP**YLPLP**RPPRPVR-NH$_2$	-1.67	11.9
67	Chex-RP**DD**PRP**YLPFP**RPPRPVR-NH$_2$	-1.73	11.9
68	Chex-RP**DD**PRP**YLPRD**RPPRPVR-NH$_2$	-2.21	11.8
69	Chex-RP**DD**PRP**YLPRK**RPPRPVR-NH$_2$	-2.23	12.2
70	Chex-RP**DD**PRP**YLPRS**RPPRPVR-NH$_2$	-2.07	12.2
71	Chex-RP**SS**PRP**DLPRP**RPPRPVR-NH$_2$	-1.94	12.8
72	Chex-RP**SS**PRP**KLPRP**RPPRPVR-NH$_2$	-1.96	13.2
73	Chex-RP**SS**PRP**SLPRP**RPPRPVR-NH$_2$	-1.8	13.2
74	Chex-RP**SS**PRP**LLPRP**RPPRPVR-NH$_2$	-1.56	13.2
75	Chex-RP**SS**PRP**YDPRP**RPPRPVR-NH$_2$	-2.21	12.5
76	Chex-RP**SS**PRP**YKPRP**RPPRPVR-NH$_2$	-2.23	12.8
77	Chex-RP**SS**PRP**YSPRP**RPPRPVR-NH$_2$	-2.07	12.8
78	Chex-RP**SS**PRP**YFPRP**RPPRPVR-NH$_2$	-1.88	12.8
79	Chex-RP**SS**PRP**YLDRP**RPPRPVR-NH$_2$	-1.93	12.5
80	Chex-RP**SS**PRP**YLKRP**RPPRPVR-NH$_2$	-1.95	12.8
81	Chex-RP**SS**PRP**YLSRP**RPPRPVR-NH$_2$	-1.78	12.8
82	Chex-RP**SS**PRP**YLPDP**RPPRPVR-NH$_2$	-1.77	12.3
83	Chex-RP**SS**PRP**YLPSP**RPPRPVR-NH$_2$	-1.63	12.7
84	Chex-RP**SS**PRP**YLPLP**RPPRPVR-NH$_2$	-1.39	12.7
85	Chex-RP**SS**PRP**YLPFP**RPPRPVR-NH$_2$	-1.44	12.7
86	Chex-RP**SS**PRP**YLPRD**RPPRPVR-NH$_2$	-1.93	12.5
87	Chex-RP**SS**PRP**YLPRK**RPPRPVR-NH$_2$	-1.95	12.8
88	Chex-RP**SS**PRP**YLPRS**RPPRPVR-NH$_2$	-1.78	12.8
89	Chex-RP**FF**PRP**DLPRP**RPPRPVR-NH$_2$	-1.56	12.8
90	Chex-RP**FF**PRP**KLPRP**RPPRPVR-NH$_2$	-1.58	13.2
91	Chex-RP**FF**PRP**SLPRP**RPPRPVR-NH$_2$	-1.42	13.2
92	Chex-RP**FF**PRP**LLPRP**RPPRPVR-NH$_2$	-1.18	13.2
93	Chex-RP**FF**PRP**YDPRP**RPPRPVR-NH$_2$	-1.83	12.5
94	Chex-RP**FF**PRP**YKPRP**RPPRPVR-NH$_2$	-1.85	12.8
95	Chex-RP**FF**PRP**YSPRP**RPPRPVR-NH$_2$	-1.69	12.8
96	Chex-RP**FF**PRP**YFPRP**RPPRPVR-NH$_2$	-1.5	12.8
97	Chex-RP**FF**PRP**YLDRP**RPPRPVR-NH$_2$	-1.55	12.5
98	Chex-RP**FF**PRP**YLKRP**RPPRPVR-NH$_2$	-1.57	12.8
99	Chex-RP**FF**PRP**YLSRP**RPPRPVR-NH$_2$	-1.41	12.8
100	Chex-RP**FF**PRP**YLPDP**RPPRPVR-NH$_2$	-1.39	12.3

Continuation of Table S1

#	Sequence	GRAVY	pI
101	Chex-RP**FF**PRP**YLPSP**RPPRPVR-NH$_2$	-1.25	12.7
102	Chex-RP**FF**PRP**YLPLP**RPPRPVR-NH$_2$	-1.01	12.7
103	Chex-RP**FF**PRP**YLPFP**RPPRPVR-NH$_2$	-1.06	12.7
104	Chex-RP**FF**PRP**YLPRD**RPPRPVR-NH$_2$	-1.55	12.5
105	Chex-RP**FF**PRP**YLPRK**RPPRPVR-NH$_2$	-1.57	12.8
106	Chex-RP**FF**PRP**YLPRS**RPPRPVR-NH$_2$	-1.41	12.8
107	Chex-RP**DK**PRP**DDPRP**RPPRPVR-NH$_2$	-2.63	12.2
108	Chex-RP**DK**PRP**DKPRP**RPPRPVR-NH$_2$	-2.65	12.5
109	Chex-RP**DK**PRP**DSPRP**RPPRPVR-NH$_2$	-2.49	12.5
110	Chex-RP**DK**PRP**DFPRP**RPPRPVR-NH$_2$	-2.3	12.5
111	Chex-RP**DK**PRP**DLDRP**RPPRPVR-NH$_2$	-2.35	12.2
112	Chex-RP**DK**PRP**DLKRP**RPPRPVR-NH$_2$	-2.37	12.5
113	Chex-RP**DK**PRP**DLPDP**RPPRPVR-NH$_2$	-2.19	11.9
114	Chex-RP**DK**PRP**DLPSP**RPPRPVR-NH$_2$	-2.05	12.3
115	Chex-RP**DK**PRP**DLPLP**RPPRPVR-NH$_2$	-1.81	12.3
116	Chex-RP**DK**PRP**DLPFP**RPPRPVR-NH$_2$	-1.86	12.3
117	Chex-RP**DK**PRP**DLPRD**RPPRPVR-NH$_2$	-2.35	12.2
118	Chex-RP**DK**PRP**DLPRK**RPPRPVR-NH$_2$	-2.37	12.5
119	Chex-RP**DK**PRP**DLPRS**RPPRPVR-NH$_2$	-2.21	12.5
120	Chex-RP**DK**PRP**KDPRP**RPPRPVR-NH$_2$	-2.65	12.5
121	Chex-RP**DK**PRP**KKPRP**RPPRPVR-NH$_2$	-2.67	12.8
122	Chex-RP**DK**PRP**KSPRP**RPPRPVR-NH$_2$	-2.51	12.8
123	Chex-RP**DK**PRP**KFPRP**RPPRPVR-NH$_2$	-2.32	12.8
124	Chex-RP**DK**PRP**SDPRP**RPPRPVR-NH$_2$	-2.09	12.5
125	Chex-RP**DK**PRP**SKPRP**RPPRPVR-NH$_2$	-2.51	12.8
126	Chex-RP**DK**PRP**SSPRP**RPPRPVR-NH$_2$	-2.35	12.8
127	Chex-RP**DK**PRP**SFPRP**RPPRPVR-NH$_2$	-2.16	12.8
128	Chex-RP**DK**PRP**SLDRP**RPPRPVR-NH$_2$	-2.21	12.5
129	Chex-RP**DK**PRP**SLKRP**RPPRPVR-NH$_2$	-2.23	12.8
130	Chex-RP**DK**PRP**SLPFP**RPPRPVR-NH$_2$	-1.72	12.7
131	Chex-RP**DK**PRP**SLPRD**RPPRPVR-NH$_2$	-2.21	12.5
132	Chex-RP**DK**PRP**SLPRK**RPPRPVR-NH$_2$	-2.23	12.8
133	Chex-RP**DK**PRP**SLPRS**RPPRPVR-NH$_2$	-2.06	12.8
134	Chex-RP**DK**PRP**LDPRP**RPPRPVR-NH$_2$	-2.25	12.5
135	Chex-RP**DK**PRP**LKPRP**RPPRPVR-NH$_2$	-2.27	12.8
136	Chex-RP**DK**PRP**LSPRP**RPPRPVR-NH$_2$	-2.11	12.8
137	Chex-RP**DK**PRP**LFPRP**RPPRPVR-NH$_2$	-1.92	12.8
138	Chex-RP**DK**PRP**LLDRP**RPPRPVR-NH$_2$	-1.96	12.5
139	Chex-RP**DK**PRP**LLKRP**RPPRPVR-NH$_2$	-1.98	12.8
140	Chex-RP**DK**PRP**LLSRP**RPPRPVR-NH$_2$	-1.82	12.8
141	Chex-RP**DK**PRP**LSPDP**RPPRPVR-NH$_2$	-2.05	12.3
142	Chex-RP**DK**PRP**LSPSP**RPPRPVR-NH$_2$	-1.91	12.7
143	Chex-RP**DK**PRP**LSPLP**RPPRPVR-NH$_2$	-1.67	12.7
144	Chex-RP**DK**PRP**LSPFP**RPPRPVR-NH$_2$	-1.72	12.7
145	Chex-RP**DK**PRP**LSPRK**RPPRPVR-NH$_2$	-2.23	12.8
146	Chex-RP**DK**PRP**LSPRS**RPPRPVR-NH$_2$	-2.06	12.8
147	Chex-RP**DK**PRP**YDDRP**RPPRPVR-NH$_2$	-2.62	11.8
148	Chex-RP**DK**PRP**YDKRP**RPPRPVR-NH$_2$	-2.64	12.2
149	Chex-RP**DK**PRP**YDSRP**RPPRPVR-NH$_2$	-2.47	12.2
150	Chex-RP**DK**PRP**YDPDP**RPPRPVR-NH$_2$	-2.46	11.2
151	Chex-RP**DK**PRP**YDPSP**RPPRPVR-NH$_2$	-2.32	11.9
152	Chex-RP**DK**PRP**YDPLP**RPPRPVR-NH$_2$	-2.08	11.9
153	Chex-RP**DK**PRP**YDPFP**RPPRPVR-NH$_2$	-2.13	11.9
154	Chex-RP**DK**PRP**YDPRD**RPPRPVR-NH$_2$	-2.62	11.8
155	Chex-RP**DK**PRP**YDPRK**RPPRPVR-NH$_2$	-2.64	12.2
156	Chex-RP**DK**PRP**YDPRS**RPPRPVR-NH$_2$	-2.47	12.2
157	Chex-RP**DK**PRP**YKDRP**RPPRPVR-NH$_2$	-2.64	12.2
158	Chex-RP**DK**PRP**YKKRP**RPPRPVR-NH$_2$	-2.66	12.5
159	Chex-RP**DK**PRP**YKSRP**RPPRPVR-NH$_2$	-2.49	12.5
160	Chex-RP**DK**PRP**YKPDP**RPPRPVR-NH$_2$	-2.48	11.9
161	Chex-RP**DK**PRP**YKPSP**RPPRPVR-NH$_2$	-2.34	12.3
162	Chex-RP**DK**PRP**YKPLP**RPPRPVR-NH$_2$	-2.1	12.3
163	Chex-RP**DK**PRP**YKPFP**RPPRPVR-NH$_2$	-2.15	12.3
164	Chex-RP**DK**PRP**YKPRD**RPPRPVR-NH$_2$	-2.64	12.2
165	Chex-RP**DK**PRP**YKPRK**RPPRPVR-NH$_2$	-2.66	12.5
166	Chex-RP**DK**PRP**YKPRS**RPPRPVR-NH$_2$	-2.49	12.5
167	Chex-RP**DK**PRP**YSDRP**RPPRPVR-NH$_2$	-2.47	12.2
168	Chex-RP**DK**PRP**YSKRP**RPPRPVR-NH$_2$	-2.49	12.5
169	Chex-RP**DK**PRP**YSSRP**RPPRPVR-NH$_2$	-2.33	12.5
170	Chex-RP**DK**PRP**YSPDP**RPPRPVR-NH$_2$	-2.32	11.9
171	Chex-RP**DK**PRP**YSPSP**RPPRPVR-NH$_2$	-2.18	12.3
172	Chex-RP**DK**PRP**YSPLP**RPPRPVR-NH$_2$	-1.94	12.3
173	Chex-RP**DK**PRP**YSPFP**RPPRPVR-NH$_2$	-1.99	12.3
174	Chex-RP**DK**PRP**YSPRD**RPPRPVR-NH$_2$	-2.47	12.2
175	Chex-RP**DK**PRP**YSPRK**RPPRPVR-NH$_2$	-2.49	12.5
176	Chex-RP**DK**PRP**YSPRS**RPPRPVR-NH$_2$	-2.33	12.5
177	Chex-RP**DK**PRP**YFDRP**RPPRPVR-NH$_2$	-2.28	12.2
178	Chex-RP**DK**PRP**YFKRP**RPPRPVR-NH$_2$	-2.31	12.5
179	Chex-RP**DK**PRP**YFSRP**RPPRPVR-NH$_2$	-2.14	12.5
180	Chex-RP**DK**PRP**YFPDP**RPPRPVR-NH$_2$	-2.13	11.9
181	Chex-RP**DK**PRP**YFPSP**RPPRPVR-NH$_2$	-1.99	12.3
182	Chex-RP**DK**PRP**YFPLP**RPPRPVR-NH$_2$	-1.75	12.3
183	Chex-RP**DK**PRP**YFPRK**RPPRPVR-NH$_2$	-2.31	12.5

Table S2: Z'-values calculated for the established high-throughput screening assay (1). The FP-based binding assay used as negative control a sample without DnaK but Cf-ARV-1502 (minimum) and as a positive control a sample with DnaK and Cf-ARV-1502 but no unlabeled peptide (maximum). The ATPase activity assay relied on a sample lacking chaperones and ATP as negative control (minimum) and a sample containing the most stimulating peptide **96** (*E. coli*) and **10** (*S. aureus*), chaperones and ATP (maximum). For the refolding activity assay samples lacking a chaperone were used as negative control (minimum) and samples lacking a peptide were used as positive control (maximum). All control samples were measured in six replicates per assay.

**Assay Type**	**Binding Strength**		**ATPase Activity**		**Refolding Activity**	
**Chaperone variant**	*E. coli*	*S. aureus*	*E. coli*	*S. aureus*	*E. coli*	*S. aureus*
**Z' factor**	0.90	0.78	0.81	0.79	0.73	0.74

Table S3: Data of obtained in all performed assays.

#	Sequence	Normalized Binding Strength		ATPase activity (%)		Refolding activity (%)		MIC (µg/mL)	
		E. coli	***S. aureus***	***E. coli***	***S. aureus***	***E. coli***	***S. aureus***	***E. coli***	***S. aureus***
1	Chex-RP**DK**PRP**YLPRP**RPPRPVR-NH_2	1.00	1.00	62.7 ± 6.5	165.3 ± 10.2	71.1 ± 5.1	112.8 ± 11.0	8	>128
2	Chex-RP**KK**PRP**YLPRP**RPPRPVR-NH_2	1.04	1.00	81.8 ± 5.6	161.6 ± 12.1	69.8 ± 1.3	115.8 ± 4.8	4	32
3	Chex-RP**SK**PRP**YLPRP**RPPRPVR-NH_2	1.03	0.99	80.0 ± 9.2	175.7 ± 9.6	67.7 ± 5.5	113.4 ± 8.6	n.d.	128
4	Chex-RP**LK**PRP**YLPRP**RPPRPVR-NH_2	1.03	0.99	62.9 ± 1.0	141.2 ± 8.9	65.6 ± 5.1	120.5 ± 6.1	16	32
5	Chex-RP**FK**PRP**YLPRP**RPPRPVR-NH_2	1.07	0.95	64.9 ± 7.6	128.2 ± 9.2	64.6 ± 3.8	110.2 ± 10.5	n.d.	32
6	Chex-RP**DD**PRP**YLPRP**RPPRPVR-NH_2	1.11	1.06	52.8 ± 1.5	164.6 ± 0.4	72.9 ± 4.1	130.0 ± 5.1	128	>128
7	Chex-RP**DS**PRP**YLPRP**RPPRPVR-NH_2	1.12	1.02	63.5 ± 6.0	165.2 ± 8.5	75.8 ± 2.0	114.1 ± 9.4	32-64	>128
8	Chex-RP**DL**PRP**YLPRP**RPPRPVR-NH_2	1.06	1.01	69.9 ± 6.4	180.0 ± 5.6	60.7 ± 0.0	126.0 ± 12.2	n.d.	n.d.
9	Chex-RP**DF**PRP**YLPRP**RPPRPVR-NH_2	1.06	1.02	51.5 ± 3.5	147.2 ± 19.7	68.0 ± 2.1	116.5 ± 8.1	n.d.	n.d.
10	Chex-RP**DK**PRP**DLPRP**RPPRPVR-NH_2	1.23	1.12	51.5 ± 5.4	218.0 ± 6.5	76.0 ± 1.5	135.7 ± 7.7	>128	>128
11	Chex-RP**DK**PRP**KLPRP**RPPRPVR-NH_2	1.12	1.06	67.8 ± 5.0	170.0 ± 16.1	73.9 ± 3.1	127.2 ± 14.3	32	>128
12	Chex-RP**DK**PRP**SLPRP**RPPRPVR-NH_2	1.11	1.01	72.0 ± 1.5	136.3 ± 1.9	73.7 ± 2.1	125.4 ± 1.9	128	>128
13	Chex-RP**DK**PRP**LLPRP**RPPRPVR-NH_2	1.00	0.98	76.0 ± 4.4	171.7 ± 11.4	67.1 ± 0.2	114.2 ± 15.5	128	>128
14	Chex-RP**DK**PRP**YDPRP**RPPRPVR-NH_2	1.25	1.13	52.2 ± 3.3	190.6 ± 5.8	73.5 ± 2.3	129.2 ± 6.2	>128	>128
15	Chex-RP**DK**PRP**YKPRP**RPPRPVR-NH_2	1.27	1.13	65.8 ± 4.4	176.2 ± 17.3	76.0 ± 1.2	117.3 ± 15.3	32	>128
16	Chex-RP**DK**PRP**YSPRP**RPPRPVR-NH_2	1.26	1.14	68.6 ± 2.2	154.8 ± 3.1	77.8 ± 3.8	122.0 ± 5.1	64	>128
17	Chex-RP**DK**PRP**YFPRP**RPPRPVR-NH_2	1.19	1.09	72.8 ± 1.2	168.4 ± 12.4	66.6 ± 3.5	124.2 ± 10.7	32	>128
18	Chex-RP**DK**PRP**YLDRP**RPPRPVR-NH_2	1.20	1.13	54.9 ± 0.5	188.3 ± 15.6	71.4 ± 5.2	131.6 ± 2.4	>128	>128
19	Chex-RP**DK**PRP**YLKRP**RPPRPVR-NH_2	1.23	1.13	69.6 ± 0.5	160.5 ± 14.2	62.7 ± 3.7	116.5 ± 10.0	32	64-128
20	Chex-RP**DK**PRP**YLSRPR**PPRPVR-NH_2	1.20	1.12	59.6 ± 6.9	161.1 ± 12.8	67.9 ± 1.5	126.9 ± 6.8	128	>128
21	Chex-RP**DK**PRP**YLPDP**RPPRPVR-NH_2	1.23	1.10	55.6 ± 2.0	171.8 ± 7.8	57.2 ± 4.7	120.8 ± 12.2	>128	>128
22	Chex-RP**DK**PRP**YLPSP**RPPRPVR-NH_2	1.16	1.03	64.4 ± 3.4	177.9 ± 10.7	63.6 ± 3.6	125.1 ± 6.3	>128	>128
23	Chex-RP**DK**PRP**YLPLP**RPPRPVR-NH_2	1.05	0.96	68.3 ± 4.7	176.9 ± 8.8	60.2 ± 3.9	124.5 ± 7.9	>128	>128
24	Chex-RP**DK**PRP**YLPFP**RPPRPVR-NH_2	1.15	1.02	57.4 ± 2.5	157.1 ± 7.6	61.9 ± 1.5	121.1 ± 4.7	>128	>128
25	Chex-RP**DK**PRP**YLPRD**RPPRPVR-NH_2	1.16	1.12	57.4 ± 4.0	175.6 ± 14.4	70.6 ± 4.2	117.6 ± 3.4	>128	>128
26	Chex-RP**DK**PRP**YLPRK**RPPRPVR-NH_2	1.15	1.08	83.5 ± 2.6	123.4 ± 5.1	69.5 ± 2.8	129.1 ± 6.0	64	128
27	Chex-RP**DK**PRP**YLPRS**RPPRPVR-NH_2	1.11	1.04	59.1 ± 5.4	156.2 ± 8.3	70.5 ± 3.6	116.5 ± 5.5	≥128	>128
28	Chex-RP**KD**PRP**YLPRP**RPPRPVR-NH_2	1.10	0.99	81.0 ± 8.6	141.2 ± 16.0	68.1 ± 9.1	106.5 ± 25.8	16	>128
29	Chex-RP**KS**PRP**YLPRP**RPPRPVR-NH_2	1.01	0.98	90.8 ± 9.6	158.6 ± 28.1	62.3 ± 1.3	108.3 ± 15.1	16	64
30	Chex-RP**KL**PRP**YLPRP**RPPRPVR-NH_2	1.00	0.98	109.2 ± 10.1	123.2 ± 10.7	64.7 ± 2.0	119.0 ± 3.0	16	32
31	Chex-RP**KF**PRP**YLPRP**RPPRPVR-NH_2	0.94	0.98	78.4 ± 4.1	127.4 ± 13.3	61.2 ± 4.4	122.1 ± 5.7	16-32	16-32
32	Chex-RP**KK**PRP**DLPRP**RPPRPVR-NH_2	1.22	1.11	70.3 ± 5.7	153.4 ± 9.0	71.9 ± 6.0	134.3 ± 3.5	64-128	>128
33	Chex-RP**KK**PRP**KLPRP**RPPRPVR-NH_2	1.05	1.02	99.0 ± 9.8	147.9 ± 14.3	68.4 ± 4.5	125.6 ± 9.7	16-32	32
34	Chex-RP**KK**PRP**SLPRP**RPPRPVR-NH_2	1.07	0.96	73.8 ± 4.5	138.0 ± 16.6	70.9 ± 1.9	129.3 ± 3.8	32	32-64
35	Chex-RP**KK**PRP**LLPRP**RPPRPVR-NH_2	1.07	0.94	84.3 ± 9.6	150.5 ± 7.8	67.5 ± 3.5	126.2 ± 7.6	32	32
36	Chex-RP**KK**PRP**YDPRP**RPPRPVR-NH_2	1.23	1.12	57.4 ± 3.3	150.6 ± 9.4	73.8 ± 4.6	131.5 ± 8.0	64	n.d.
37	Chex-RP**KK**PRP**YKPRP**RPPRPVR-NH_2	1.24	1.10	70.6 ± 2.3	169.5 ± 1.8	64.8 ± 3.0	121.1 ± 5.7	16	32
38	Chex-RP**KK**PRP**YSPRP**RPPRPVR-NH_2	1.23	1.12	74.3 ± 8.1	160.7 ± 14.3	74.1 ± 2.9	122.0 ± 1.2	16-32	64
39	Chex-RP**KK**PRP**YFPRP**RPPRPVR-NH_2	1.15	1.03	72.3 ± 9.0	124.3 ± 0.9	63.7 ± 4.2	109.3 ± 13.6	16	16-32
40	Chex-RP**KK**PRP**YLDRP**RPPRPVR-NH_2	1.20	1.09	80.9 ± 4.9	135.7 ± 7.2	70.4 ± 1.4	126.7 ± 8.3	128->128	32

#	Sequence	Normalized Binding Strength		ATPase activity (%)		Refolding activity (%)		MIC (µg/mL)	
		E. coli	*S. aureus*	*E. coli*	*S. aureus*	*E. coli*	*S. aureus*	*E. coli*	*S. aureus*
41	Chex-RP**KK**PRP**YLKRP**RPPRPVR-NH$_2$	1.19	1.12	76.2 ± 6.6	174.7 ± 0.6	59.7 ± 1.0	119.6 ± 1.5	64-128	32-64
42	Chex-RP**KK**PRP**YLSRP**RPPRPVR-NH$_2$	1.19	1.11	82.4 ± 3.0	142.9 ± 7.9	61.8 ± 2.2	118.7 ± 4.1	32	32
43	Chex-RP**KK**PRP**YLPDP**RPPRPVR-NH$_2$	1.20	1.06	77.2 ± 4.3	168.6 ± 9.2	60.8 ± 3.4	113.5 ± 11.6	>128	>128
44	Chex-RP**KK**PRP**YLPSP**RPPRPVR-NH$_2$	1.07	0.98	71.4 ± 3.8	170.1 ± 13.9	61.9 ± 1.3	119.7 ± 12.0	32	64
45	Chex-RP**KK**PRP**YLPLP**RPPRPVR-NH$_2$	0.97	0.91	74.5 ± 10.7	78.0 ± 7.0	60.7 ± 4.9	106.0 ± 16.3	16-32	32-64
46	Chex-RP**KK**PRP**YLPFP**RPPRPVR-NH$_2$	1.14	0.96	91.2 ± 11.0	130.9 ± 6.7	56.9 ± 1.6	115.8 ± 4.3	16	32
47	Chex-RP**KK**PRP**YLPRD**RPPRPVR-NH$_2$	1.20	1.12	76.8 ± 5.2	140.7 ± 12.9	65.7 ± 2.6	114.0 ± 16.4	64	64
48	Chex-RP**KK**PRP**YLPRK**RPPRPVR-NH$_2$	1.14	1.05	71.3 ± 10.7	133.1 ± 5.1	62.6 ± 2.8	115.3 ± 5.9	32	32
49	Chex-RP**KK**PRP**YLPRS**RPPRPVR-NH$_2$	1.13	1.03	69.5 ± 5.4	178.8 ± 6.3	61.3 ± 3.2	108.9 ± 9.8	32	32
50	Chex-RP**SD**PRP**YLPRP**RPPRPVR-NH$_2$	1.04	1.02	80.1 ± 3.5	156.0 ± 4.7	71.6 ± 0.9	121.9 ± 8.3	128	>128
51	Chex-RP**LD**PRP**YLPRP**RPPRPVR-NH$_2$	1.01	0.98	54.7 ± 4.3	147.4 ± 18.5	60.9 ± 0.5	114.3 ± 5.4	128	>128
52	Chex-RP**FD**PRP**YLPRP**RPPRPVR-NH$_2$	1.02	1.00	57.0 ± 3.5	121.1 ± 3.7	60.5 ± 1.9	108.2 ± 9.3	64-128	64-128
53	Chex-RP**DD**PRP**DLPRP**RPPRPVR-NH$_2$	1.27	1.12	78.5 ± 5.0	166.1 ± 10.0	68.0 ± 4.6	111.3 ± 10.9	>128	>128
54	Chex-RP**DD**PRP**KLPRP**RPPRPVR-NH$_2$	1.12	1.07	87.5 ± 6.2	112.2 ± 2.0	66.1 ± 4.8	129.5 ± 7.7	>128	>128
55	Chex-RP**DD**PRP**SLPRP**RPPRPVR-NH$_2$	1.09	1.05	74.6 ± 6.1	151.4 ± 3.4	64.9 ± 2.4	117.8 ± 14.5	>128	>128
56	Chex-RP**DD**PRP**LLPRP**RPPRPVR-NH$_2$	1.04	0.99	52.9 ± 8.5	112.6 ± 7.9	65.3 ± 2.5	131.8 ± 6.2	>128	>128
57	Chex-RP**DD**PRP**YDPRP**RPPRPVR-NH$_2$	1.25	1.11	68.0 ± 4.1	157.6 ± 19.8	66.5 ± 1.0	123.8 ± 4.1	>128	>128
58	Chex-RP**DD**PRP**YKPRP**RPPRPVR-NH$_2$	1.27	1.10	75.7 ± 0.3	172.8 ± 9.9	73.4 ± 3.9	131.2 ± 4.5	128	>128
59	Chex-RP**DD**PRP**YSPRP**RPPRPVR-NH$_2$	1.24	1.11	77.7 ± 2.4	159.4 ± 8.7	67.5 ± 5.4	112.1 ± 19.6	>128	>128
60	Chex-RP**DD**PRP**YFPRP**RPPRPVR-NH$_2$	1.18	1.07	50.6 ± 5.1	119.6 ± 2.6	67.5 ± 2.8	116.2 ± 9.8	128	>128
61	Chex-RP**DD**PRP**YLDRP**RPPRPVR-NH$_2$	1.19	1.12	56.5 ± 3.7	144.0 ± 10.0	65.5 ± 0.5	112.2 ± 16.0	>128	>128
62	Chex-RP**DD**PRP**YLKRP**RPPRPVR-NH$_2$	1.26	1.14	63.2 ± 3.7	143.9 ± 8.4	65.2 ± 3.8	113.6 ± 12.4	128	>128
63	Chex-RP**DD**PRP**YLSRP**RPPRPVR-NH$_2$	1.20	1.10	73.6 ± 3.4	135.8 ± 4.5	64.4 ± 1.6	107.1 ± 12.9	>128	>128
64	Chex-RP**DD**PRP**YLPDP**RPPRPVR-NH$_2$	1.24	1.07	48.4 ± 4.9	166.0 ± 7.7	63.2 ± 0.8	119.6 ± 6.0	>128	>128
65	Chex-RP**DD**PRP**YLPSP**RPPRPVR-NH$_2$	1.08	0.99	71.6 ± 4.5	171.1 ± 11.2	61.5 ± 0.4	111.4 ± 16.1	>128	>128
66	Chex-RP**DD**PRP**YLPLP**RPPRPVR-NH$_2$	1.03	0.96	41.3 ± 5.6	159.3 ± 13.1	58.0 ± 2.6	123.2 ± 4.0	>128	>128
67	Chex-RP**DD**PRP**YLPFP**RPPRPVR-NH$_2$	1.13	1.00	56.9 ± 3.3	160.7 ± 7.1	58.8 ± 3.0	98.9 ± 9.9	>128	>128
68	Chex-RP**DD**PRP**YLPRD**RPPRPVR-NH$_2$	1.21	1.13	44.8 ± 5.6	134.1 ± 5.9	62.2 ± 4.9	112.6 ± 13.9	>128	>128
69	Chex-RP**DD**PRP**YLPRK**RPPRPVR-NH$_2$	1.16	1.10	71.5 ± 4.6	156.6 ± 8.0	60.6 ± 3.0	101.4 ± 13.0	>128	>128
70	Chex-RP**DD**PRP**YLPRS**RPPRPVR-NH$_2$	1.13	1.10	57.2 ± 3.8	125.2 ± 12.2	63.0 ± 1.3	108.8 ± 11.9	>128	>128
71	Chex-RP**SS**PRP**DLPRP**RPPRPVR-NH$_2$	1.22	1.11	85.9 ± 6.4	134.8 ± 8.2	63.4 ± 1.3	104.8 ± 9.2	>128	>128
72	Chex-RP**SS**PRP**KLPRP**RPPRPVR-NH$_2$	1.10	1.03	96.3 ± 17.4	154.9 ± 8.0	60.5 ± 0.9	89.2 ± 7.1	64	128
73	Chex-RP**SS**PRP**SLPRP**RPPRPVR-NH$_2$	1.04	1.01	88.8 ± 6.9	164.7 ± 15.3	61.3 ± 0.2	122.8 ± 0.7	128	>128
74	Chex-RP**SS**PRP**LLPRP**RPPRPVR-NH$_2$	0.99	0.99	85.5 ± 12.7	142.9 ± 10.4	63.9 ± 6.1	129.1 ± 12.6	64	>128
75	Chex-RP**SS**PRP**YDPRP**RPPRPVR-NH$_2$	1.22	1.10	81.7 ± 4.9	158.9 ± 8.4	63.8 ± 0.6	131.8 ± 8.6	>128	>128
76	Chex-RP**SS**PRP**YKPRP**RPPRPVR-NH$_2$	1.23	1.12	82.4 ± 11.7	146.0 ± 5.0	62.8 ± 6.3	124.3 ± 5.2	16-32	64
77	Chex-RP**SS**PRP**YSPRP**RPPRPVR-NH$_2$	1.21	1.10	101.9 ± 11.6	150.8 ± 6.6	55.1 ± 2.7	122.0 ± 3.5	64	>128
78	Chex-RP**SS**PRP**YFPRP**RPPRPVR-NH$_2$	1.16	1.04	83.3 ± 13.1	110.7 ± 1.2	44.9 ± 4.8	125.8 ± 5.2	16	64
79	Chex-RP**SS**PRP**YLDRP**RPPRPVR-NH$_2$	1.15	1.11	90.3 ± 10.7	147.1 ± 9.6	62.0 ± 1.3	127.1 ± 1.7	>128	>128
80	Chex-RP**SS**PRP**YLKRP**RPPRPVR-NH$_2$	1.19	1.15	70.5 ± 9.4	114.2 ± 9.6	62.2 ± 7.5	122.7 ± 4.3	32	32
81	Chex-RP**SS**PRP**YLSRP**RPPRPVR-NH$_2$	1.16	1.09	85.3 ± 8.4	139.1 ± 4.7	59.1 ± 2.9	117.5 ± 3.6	128	64-128
82	Chex-RP**SS**PRP**YLPDP**RPPRPVR-NH$_2$	1.21	1.07	66.1 ± 3.9	107.7 ± 2.1	61.6 ± 3.3	122.0 ± 4.7	>128	>128
83	Chex-RP**SS**PRP**YLPSP**RPPRPVR-NH$_2$	1.04	0.97	97.1 ± 8.7	180.2 ± 20.7	56.4 ± 1.1	121.9 ± 4.4	>128	>128
84	Chex-RP**SS**PRP**YLPLP**RPPRPVR-NH$_2$	1.01	0.92	79.9 ± 2.1	150.5 ± 3.4	60.6 ± 3.4	123.8 ± 0.9	>128	>128

#	Sequence	Normalized Binding Strength		ATPase activity (%)		Refolding activity (%)		MIC (µg/mL)	
		E. coli	*S. aureus*	*E. coli*	*S. aureus*	*E. coli*	*S. aureus*	*E. coli*	*S. aureus*
85	Chex-RPSSPRPYLPFPRPPRPVR-NH$_2$	1.05	0.94	70.3 ± 5.0	127.9 ± 4.1	54.6 ± 2.1	115.8 ± 6.9	128	>128
86	Chex-RPSSPRPYLPRDRPPRPVR-NH$_2$	1.18	1.13	72.6 ± 10.1	126.3 ± 8.5	60.3 ± 2.8	125.2 ± 4.6	>128	>128
87	Chex-RPSSPRPYLPRKRPPRPVR-NH$_2$	1.12	1.05	89.4 ± 4.1	128.0 ± 3.8	47.0 ± 3.8	122.3 ± 4.1	64	64
88	Chex-RPSSPRPYLPRSRPPRPVR-NH$_2$	1.11	1.07	91.3 ± 10.6	129.8 ± 6.4	50.7 ± 3.3	119.2 ± 2.5	128	128
89	Chex-RPFFPRPDLPRPRPPRPVR-NH$_2$	1.19	1.07	73.8 ± 2.0	119.0 ± 6.9	90.7 ± 1.5	113.6 ± 9.1	64-128	128
90	Chex-RPFFPRPKLPRPRPPRPVR-NH$_2$	1.05	1.01	100.4 ± 1.9	105.2 ± 1.4	65.3 ± 8.9	113.8 ± 4.0	16	16-32
91	Chex-RPFFPRPSLPRPRPPRPVR-NH$_2$	1.01	0.94	83.4 ± 9.5	110.4 ± 3.1	48.0 ± 2.8	106.7 ± 8.2	16-32	32
92	Chex-RPFFPRPLLPRPRPPRPVR-NH$_2$	1.11	0.95	96.0 ± 7.8	88.0 ± 13.0	45.3 ± 7.1	108.8 ± 5.5	16	16
93	Chex-RPFFPRPYDPRPRPPRPVR-NH$_2$	1.10	1.01	87.2 ± 6.0	113.4 ± 8.8	43.6 ± 2.6	106.7 ± 12.0	32	64
94	Chex-RPFFPRPYKPRPRPPRPVR-NH$_2$	1.10	1.00	93.6 ± 7.1	91.3 ± 7.3	41.6 ± 4.0	103.6 ± 7.6	16	16
95	Chex-RPFFPRPYSPRPRPPRPVR-NH$_2$	1.09	0.99	97.1 ± 7.5	103.7 ± 8.8	43.1 ± 2.4	101.0 ± 11.1	16	32
96	Chex-RPFFPRPYFPRPRPPRPVR-NH$_2$	1.16	1.02	121.7 ± 5.1	118.2 ± 15.7	36.1 ± 2.4	94.5 ± 7.7	16	32
97	Chex-RPFFPRPYLDRPRPPRPVR-NH$_2$	0.99	1.04	75.7 ± 4.4	112.7 ± 5.0	55.7 ± 2.9	98.6 ± 9.9	64-128	32
98	Chex-RPFFPRPYLKRPRPPRPVR-NH$_2$	1.06	1.06	58.7 ± 7.0	104.4 ± 12.7	51.6 ± 3.8	108.0 ± 0.9	8-16	16
99	Chex-RPFFPRPYLSRPRPPRPVR-NH$_2$	1.00	1.02	80.9 ± 7.2	112.3 ± 0.7	50.7 ± 0.8	99.3 ± 7.6	16-32	16
100	Chex-RPFFPRPYLPDPRPPRPVR-NH$_2$	1.10	1.03	59.1 ± 3.9	102.6 ± 0.5	64.6 ± 3.1	121.3 ± 5.2	≥128	>128
101	Chex-RPFFPRPYLPSPRPPRPVR-NH$_2$	1.06	0.96	100.7 ± 4.1	118.1 ± 4.7	53.2 ± 2.0	99.6 ± 5.8	128	32
102	Chex-RPFFPRPYLPLPRPPRPVR-NH$_2$	0.84	0.89	84.5 ± 12.2	83.6 ± 3.8	48.1 ± 2.8	102.3 ± 4.4	32-64	16
103	Chex-RPFFPRPYLPFPRPPRPVR-NH$_2$	1.01	0.93	69.4 ± 3.9	103.9 ± 10.1	44.5 ± 1.8	94.9 ± 8.9	32	16
104	Chex-RPFFPRPYLPRDRPPRPVR-NH$_2$	1.14	1.01	70.3 ± 0.9	93.7 ± 9.2	64.0 ± 3.6	117.9 ± 1.1	128	32
105	Chex-RPFFPRPYLPRKRPPRPVR-NH$_2$	1.03	1.02	96.7 ± 11.3	110.4 ± 10.0	46.1 ± 1.4	101.7 ± 5.8	32-64	16
106	Chex-RPFFPRPYLPRSRPPRPVR-NH$_2$	1.06	1.01	84.5 ± 4.2	73.3 ± 1.6	49.0 ± 2.8	104.7 ± 2.5	64	16
107	Chex-RPDKPRPDDPRPRPPRPVR-NH$_2$	1.26	1.16	55.3 ± 0.3	168.0 ± 12.1	71.0 ± 3.2	106.6 ± 13.3	>128	>128
108	Chex-RPDKPRPDKPRPRPPRPVR-NH$_2$	1.25	1.16	58.5 ± 3.8	135.6 ± 8.9	78.0 ± 3.3	124.8 ± 2.3	>128	>128
109	Chex-RPDKPRPDSPRPRPPRPVR-NH$_2$	1.24	1.13	58.6 ± 1.3	154.6 ± 9.0	75.8 ± 2.1	116.3 ± 9.1	>128	>128
110	Chex-RPDKPRPDFPRPRPPRPVR-NH$_2$	1.23	1.15	61.8 ± 2.4	122.0 ± 5.6	68.0 ± 4.4	120.8 ± 7.2	>128	>128
111	Chex-RPDKPRPDLDRPRPPRPVR-NH$_2$	1.24	1.12	54.6 ± 3.0	175.7 ± 9.0	48.7 ± 2.8	101.7 ± 12.5	>128	>128
112	Chex-RPDKPRPDLKRPRPPRPVR-NH$_2$	1.25	1.14	74.5 ± 4.2	141.0 ± 10.2	59.2 ± 3.0	117.3 ± 5.8	>128	>128
113	Chex-RPDKPRPDLPDPRPPRPVR-NH$_2$	1.26	1.15	53.7 ± 6.4	131.6 ± 6.7	67.0 ± 3.2	99.2 ± 1.8	>128	>128
114	Chex-RPDKPRPDLPSPRPPRPVR-NH$_2$	1.22	1.11	71.7 ± 0.7	174.9 ± 8.2	64.2 ± 0.5	106.9 ± 2.2	>128	>128
115	Chex-RPDKPRPDLPLPRPPRPVR-NH$_2$	1.20	1.10	48.2 ± 0.8	142.1 ± 9.2	60.3 ± 4.1	83.6 ± 8.4	>128	>128
116	Chex-RPDKPRPDLPFPRPPRPVR-NH$_2$	1.20	1.12	54.2 ± 1.4	151.4 ± 8.8	65.7 ± 0.6	114.2 ± 4.7	>128	>128
117	Chex-RPDKPRPDLPRDRPPRPVR-NH$_2$	1.28	1.12	47.9 ± 2.4	161.9 ± 5.5	62.4 ± 2.1	94.9 ± 4.4	>128	>128
118	Chex-RPDKPRPDLPRKRPPRPVR-NH$_2$	1.24	1.11	69.5 ± 2.0	137.1 ± 7.7	67.0 ± 4.6	109.2 ± 6.0	>128	>128
119	Chex-RPDKPRPDLPRSRPPRPVR-NH$_2$	1.25	1.12	43.7 ± 3.5	176.6 ± 7.6	64.8 ± 5.5	105.5 ± 9.2	>128	>128
120	Chex-RPDKPRPKDPRPRPPRPVR-NH$_2$	1.25	1.14	76.5 ± 3.8	140.2 ± 7.7	67.4 ± 0.9	106.5 ± 3.3	>128	>128
121	Chex-RPDKPRPKKPRPRPPRPVR-NH$_2$	1.23	1.14	56.3 ± 2.3	164.9 ± 9.1	72.9 ± 9.6	122.0 ± 11.8	32	64-128
122	Chex-RPDKPRPKSPRPRPPRPVR-NH$_2$	1.25	1.14	72.4 ± 9.1	144.3 ± 3.2	70.7 ± 2.5	119.4 ± 5.9	128	>128
123	Chex-RPDKPRPKFPRPRPPRPVR-NH$_2$	1.16	1.09	69.7 ± 5.5	119.7 ± 12.3	60.8 ± 7.3	125.2 ± 5.5	32	128
124	Chex-RPDKPRPSDPRPRPPRPVR-NH$_2$	1.24	1.06	65.5 ± 2.5	144.8 ± 8.9	67.1 ± 6.5	115.8 ± 5.5	>128	>128
125	Chex-RPDKPRPSKPRPRPPRPVR-NH$_2$	1.24	1.13	66.9 ± 5.9	109.1 ± 10.7	54.8 ± 4.0	118.2 ± 15.6	128	>128
126	Chex-RPDKPRPSSPRPRPPRPVR-NH$_2$	1.25	1.14	75.2 ± 6.3	144.3 ± 5.9	66.1 ± 2.5	114.1 ± 7.8	>128	>128
127	Chex-RPDKPRPSFPRPRPPRPVR-NH$_2$	1.23	1.06	54.6 ± 10.3	159.0 ± 8.0	58.5 ± 2.0	100.2 ± 8.1	128	>128

#	Sequence	Normalized Binding Strength		ATPase activity (%)		Refolding activity (%)		MIC (µg/mL)	
		E. coli	*S. aureus*	*E. coli*	*S. aureus*	*E. coli*	*S. aureus*	*E. coli*	*S. aureus*
128	Chex-RP**DK**PRP**SLDRP**RPPRPVR-NH_2	1.21	1.12	40.6 ± 2.8	106.0 ± 11.6	62.1 ± 0.1	110.5 ± 5.7	>128	>128
129	Chex-RP**DK**PRP**SLKRP**RPPRPVR-NH_2	1.22	1.13	59.2 ± 6.0	155.4 ± 1.0	59.7 ± 2.3	105.4 ± 5.9	64	>128
130	Chex-RP**DK**PRP**SLPFP**RPPRPVR-NH_2	1.16	1.03	53.2 ± 4.2	139.9 ± 5.5	56.0 ± 4.4	101.4 ± 12.0	>128	>128
131	Chex-RP**DK**PRP**SLPRD**RPPRPVR-NH_2	1.21	1.11	49.5 ± 3.7	149.4 ± 7.6	61.6 ± 3.6	99.4 ± 2.9	>128	>128
132	Chex-RP**DK**PRP**SLPRK**RPPRPVR-NH_2	1.17	1.09	59.8 ± 3.6	140.0 ± 4.7	65.1 ± 2.2	104.4 ± 7.7	128	>128
133	Chex-RP**DK**PRP**SLPRS**RPPRPVR-NH_2	1.15	1.09	80.6 ± 2.3	110.5 ± 5.3	62.1 ± 6.4	108.9 ± 4.2	>128	>128
134	Chex-RP**DK**PRP**LDPRP**RPPRPVR-NH_2	1.25	1.12	57.3 ± 2.2	130.9 ± 10.0	65.2 ± 5.3	109.6 ± 7.2	>128	>128
135	Chex-RP**DK**PRP**LKPRP**RPPRPVR-NH_2	1.21	1.11	73.3 ± 5.6	136.8 ± 1.7	70.0 ± 2.1	101.6 ± 3.5	32	>128
136	Chex-RP**DK**PRP**LSPRP**RPPRPVR-NH_2	1.22	1.10	77.5 ± 10.4	126.6 ± 3.1	67.3 ± 0.5	115.6 ± 4.6	128	>128
137	Chex-RP**DK**PRP**LFPRP**RPPRPVR-NH_2	1.15	1.00	59.8 ± 3.6	83.1 ± 6.6	63.7 ± 1.7	108.6 ± 4.1	64-128	128
138	Chex-RP**DK**PRP**LLDRP**RPPRPVR-NH_2	1.14	1.08	80.6 ± 2.3	120.5 ± 3.6	62.2 ± 4.3	116.8 ± 5.7	>128	>128
139	Chex-RP**DK**PRP**LLKRP**RPPRPVR-NH_2	1.15	1.08	54.2 ± 5.2	144.3 ± 12.4	64.9 ± 2.2	114.4 ± 6.9	64	128
140	Chex-RP**DK**PRP**LLSRP**RPPRPVR-NH_2	1.09	1.10	56.2 ± 3.5	131.3 ± 4.3	64.8 ± 3.0	108.8 ± 2.0	>128	>128
141	Chex-RP**DK**PRP**LSPDP**RPPRPVR-NH_2	1.24	1.12	65.2 ± 1.7	145.8 ± 8.1	63.0 ± 0.8	115.7 ± 0.3	>128	>128
142	Chex-RP**DK**PRP**LSPSP**RPPRPVR-NH_2	1.17	1.08	72.7 ± 6.2	134.9 ± 7.7	64.5 ± 2.8	122.9 ± 1.8	>128	n. d.
143	Chex-RP**DK**PRP**LSPLP**RPPRPVR-NH_2	1.19	1.09	75.4 ± 4.6	132.7 ± 1.8	64.7 ± 3.3	109.8 ± 1.1	>128	>128
144	Chex-RP**DK**PRP**LSPFP**RPPRPVR-NH_2	1.21	1.10	73.7 ± 8.5	153.6 ± 7.2	66.8 ± 1.7	115.2 ± 4.0	128	>128
145	Chex-RP**DK**PRP**LSPRK**RPPRPVR-NH_2	1.24	1.10	70.1 ± 5.5	154.1 ± 7.9	64.0 ± 1.8	109.0 ± 5.5	128	>128
146	Chex-RP**DK**PRP**LSPRS**RPPRPVR-NH_2	1.24	1.14	75.1 ± 2.3	133.5 ± 2.4	73.3 ± 2.1	117.1 ± 4.8	>128	>128
147	Chex-RP**DK**PRP**YDDRP**RPPRPVR-NH_2	1.24	1.11	66.3 ± 5.3	145.8 ± 14.3	63.3 ± 3.1	105.4 ± 4.7	128	>128
148	Chex-RP**DK**PRP**YDKRP**RPPRPVR-NH_2	1.26	1.12	77.0 ± 4.2	138.7 ± 6.2	66.1 ± 2.2	118.7 ± 2.3	>128	>128
149	Chex-RP**DK**PRP**YDSRP**RPPRPVR-NH_2	1.24	1.14	71.2 ± 1.6	148.6 ± 10.1	62.2 ± 1.5	93.9 ± 4.9	>128	>128
150	Chex-RP**DK**PRP**YDPDP**RPPRPVR-NH_2	1.24	1.13	69.0 ± 7.2	134.3 ± 2.1	57.9 ± 5.3	114.8 ± 4.7	>128	>128
151	Chex-RP**DK**PRP**YDPSP**RPPRPVR-NH_2	1.21	1.06	54.2 ± 2.7	138.1 ± 12.1	55.0 ± 2.1	98.8 ± 4.0	>128	>128
152	Chex-RP**DK**PRP**YDPLP**RPPRPVR-NH_2	1.23	1.11	73.1 ± 0.8	114.3 ± 9.0	61.5 ± 0.3	107.2 ± 2.8	>128	>128
153	Chex-RP**DK**PRP**YDPFP**RPPRPVR-NH_2	1.25	1.13	53.2 ± 3.2	145.4 ± 0.9	65.6 ± 4.2	88.8 ± 17.6	>128	>128
154	Chex-RP**DK**PRP**YDPRD**RPPRPVR-NH_2	1.24	1.12	69.2 ± 2.0	126.9 ± 8.5	63.7 ± 1.5	106.2 ± 2.8	128	>128
155	Chex-RP**DK**PRP**YDPRK**RPPRPVR-NH_2	1.24	1.11	67.2 ± 1.8	150.5 ± 9.1	61.0 ± 4.4	101.0 ± 4.5	>128	>128
156	Chex-RP**DK**PRP**YDPRS**RPPRPVR-NH_2	1.25	1.13	74.8 ± 6.6	129.4 ± 5.1	60.9 ± 2.9	104.1 ± 4.0	128	>128
157	Chex-RP**DK**PRP**YKDRP**RPPRPVR-NH_2	1.25	1.11	56.4 ± 4.0	145.9 ± 7.4	63.7 ± 6.1	97.0 ± 7.9	64	>128
158	Chex-RP**DK**PRP**YKKRP**RPPRPVR-NH_2	1.24	1.12	73.4 ± 7.0	135.1 ± 6.6	63.8 ± 5.7	96.7 ± 9.7	≥128	32
159	Chex-RP**DK**PRP**YKSRP**RPPRPVR-NH_2	1.26	1.11	58.0 ± 4.7	135.2 ± 6.7	50.2 ± 2.7	100.9 ± 2.7	64-128	>128
160	Chex-RP**DK**PRP**YKPDP**RPPRPVR-NH_2	1.23	1.10	80.3 ± 6.6	118.4 ± 2.8	53.7 ± 3.2	114.4 ± 6.2	128	>128
161	Chex-RP**DK**PRP**YKPSP**RPPRPVR-NH_2	1.11	1.06	64.6 ± 8.1	116.7 ± 0.2	56.0 ± 3.3	101.7 ± 5.8	128	>128
162	Chex-RP**DK**PRP**YKPLP**RPPRPVR-NH_2	1.21	1.11	64.1 ± 5.9	89.5 ± 4.7	55.1 ± 2.8	108.0 ± 6.9	64	>128
163	Chex-RP**DK**PRP**YKPFP**RPPRPVR-NH_2	1.24	1.12	71.3 ± 5.4	138.4 ± 10.3	59.8 ± 3.3	105.8 ± 3.3	>128	>128
164	Chex-RP**DK**PRP**YKPRD**RPPRPVR-NH_2	1.22	1.12	62.1 ± 4.9	108.1 ± 9.1	50.3 ± 5.2	110.5 ± 0.5	64	>128
165	Chex-RP**DK**PRP**YKPRK**RPPRPVR-NH_2	1.25	1.10	79.9 ± 2.3	113.0 ± 1.1	52.3 ± 3.0	99.9 ± 7.0	64-128	64
166	Chex-RP**DK**PRP**YKPRS**RPPRPVR-NH_2	1.23	1.13	52.5 ± 0.0	115.9 ± 5.1	50.0 ± 1.8	116.4 ± 3.0	>128	>128
167	Chex-RP**DK**PRP**YSDRP**RPPRPVR-NH_2	1.22	1.16	70.3 ± 1.2	118.0 ± 16.5	57.8 ± 0.4	103.1 ± 0.5	64	>128
168	Chex-RP**DK**PRP**YSKRP**RPPRPVR-NH_2	1.23	1.12	74.2 ± 6.9	120.0 ± 9.4	52.6 ± 2.3	116.4 ± 3.8	128	>128
169	Chex-RP**DK**PRP**YSSRP**RPPRPVR-NH_2	1.28	1.14	70.0 ± 1.0	128.2 ± 6.2	56.8 ± 0.2	101.2 ± 6.5	>128	>128
170	Chex-RP**DK**PRP**YSPDP**RPPRPVR-NH_2	1.23	1.08	85.4 ± 3.2	117.3 ± 5.8	52.9 ± 1.3	108.2 ± 3.5	>128	>128
171	Chex-RP**DK**PRP**YSPSP**RPPRPVR-NH_2	1.16	1.05	61.5 ± 8.8	116.4 ± 4.8	56.8 ± 3.9	101.2 ± 1.3	>128	>128

#	Sequence	Normalized Binding Strength		ATPase activity (%)		Refolding activity (%)		MIC (µg/mL)	
		E. coli	***S. aureus***	***E. coli***	***S. aureus***	***E. coli***	***S. aureus***	***E. coli***	***S. aureus***
172	Chex-RP**DK**PRP**YSPLP**RPPRPVR-NH$_2$	1.22	1.09	90.9 ± 10.0	89.0 ± 5.1	51.6 ± 1.8	105.1 ± 3.8	>128	>128
173	Chex-RP**DK**PRP**YSPFP**RPPRPVR-NH$_2$	1.24	1.12	71.5 ± 1.7	129.6 ± 3.9	61.8 ± 1.4	99.3 ± 7.5	>128	>128
174	Chex-RP**DK**PRP**YSPRD**RPPRPVR-NH$_2$	1.24	1.11	73.4 ± 8.6	97.3 ± 2.8	44.7 ± 0.4	107.3 ± 4.9	64	>128
175	Chex-RP**DK**PRP**YSPRK**RPPRPVR-NH$_2$	1.25	1.10	81.8 ± 7.0	163.3 ± 13.5	53.5 ± 1.7	96.5 ± 11.2	>128	>128
176	Chex-RP**DK**PRP**YSPRS**RPPRPVR-NH$_2$	1.21	1.11	74.4 ± 8.7	106.2 ± 4.7	45.2 ± 2.6	103.0 ± 1.2	128	>128
177	Chex-RP**DK**PRP**YFDRP**RPPRPVR-NH$_2$	1.22	1.10	51.0 ± 3.9	114.7 ± 2.7	54.2 ± 0.3	86.8 ± 4.2	32	128
178	Chex-RP**DK**PRP**YFKRP**RPPRPVR-NH$_2$	1.23	1.12	71.3 ± 8.7	107.8 ± 1.3	46.9 ± 2.1	101.6 ± 3.6	128	32
179	Chex-RP**DK**PRP**YFSRP**RPPRPVR-NH$_2$	1.22	1.07	63.8 ± 1.9	123.2 ± 2.5	54.8 ± 3.1	93.6 ± 8.6	>128	128
180	Chex-RP**DK**PRP**YFPDP**RPPRPVR-NH$_2$	1.16	1.04	74.0 ± 9.4	107.5 ± 1.4	41.9 ± 2.5	99.7 ± 10.0	>128	>128
181	Chex-RP**DK**PRP**YFPSP**RPPRPVR-NH$_2$	1.08	0.99	57.8 ± 2.7	118.7 ± 3.3	56.4 ± 0.2	89.2 ± 4.1	>128	>128
182	Chex-RP**DK**PRP**YFPLP**RPPRPVR-NH$_2$	1.18	1.05	70.3 ± 8.4	104.8 ± 5.8	38.2 ± 3.2	100.2 ± 4.7	>128	>128
183	Chex-RP**DK**PRP**YFPRK**RPPRPVR-NH$_2$	1.17	1.08	69.2 ± 2.2	107.7 ± 4.3	63.6 ± 1.5	84.6 ± 2.0	64-128	>128

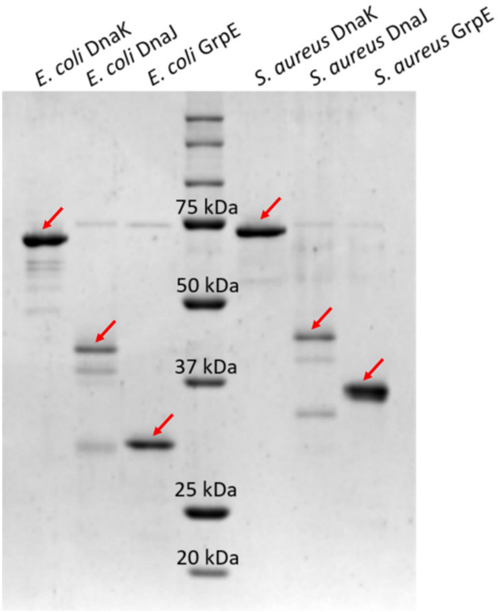

Figure S1: SDS-PAGE of purified recombinant chaperones and co-chaperones as indicated on top. The bands marked with an arrow contained the corresponding proteins as confirmed by LC-MS after tryptic in-gel digestion.

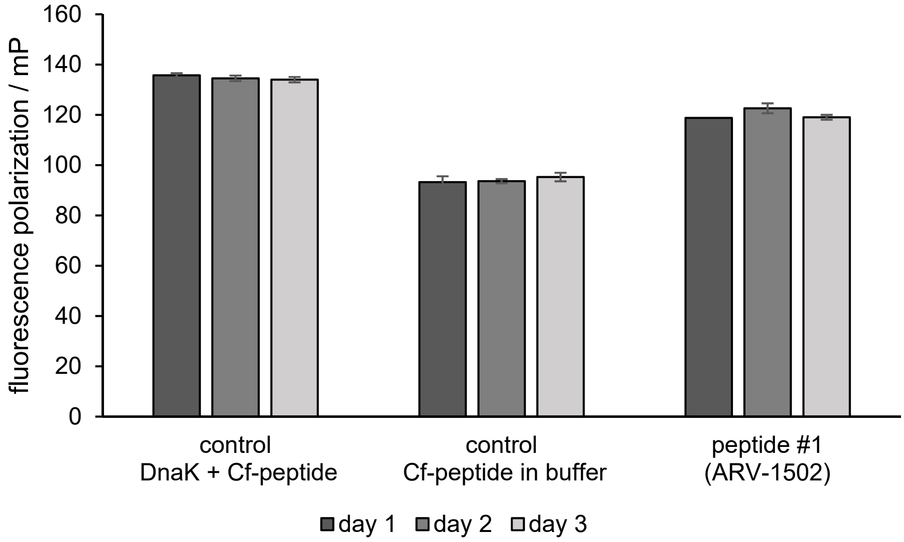

Figure S2: The optimized FP-assay provided highly reproducible data recorded for *E. coli* DnaK and Cf-ARV-1502 on three consecutive days in triplicates.

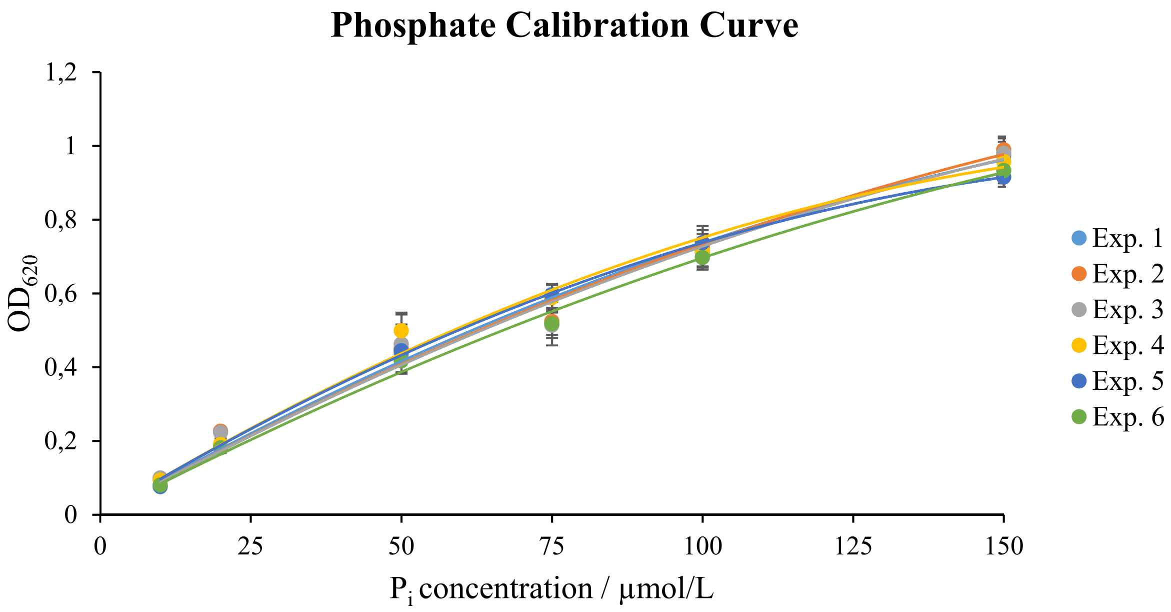

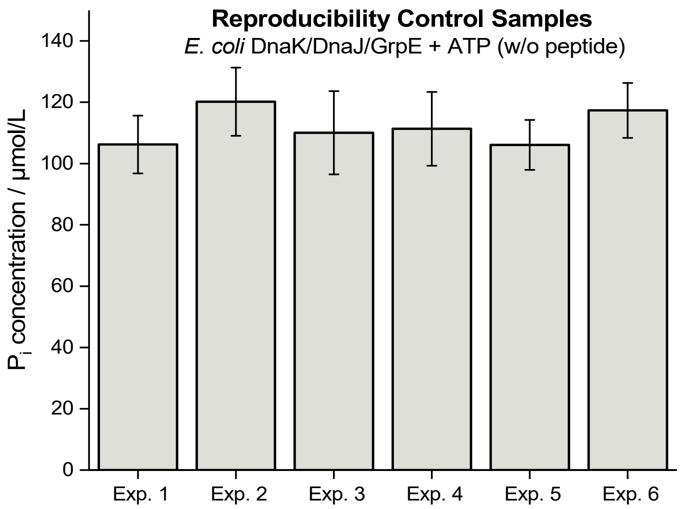

Figure S3: Validation of ATPase activity assay. Phosphate calibration curve (top) and control samples with *E. coli* DnaK/DnaJ/GrpE and ATP (bottom) were measured on two different plates in triplicates for three consecutive days.

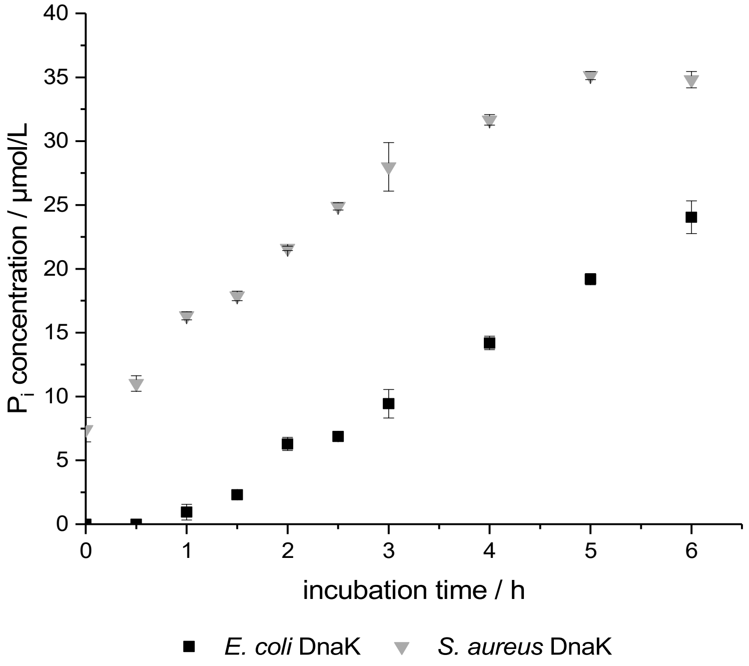

Figure S4: ATPase activity of in-house expressed and purified *E. coli* and *S. aureus* DnaK.

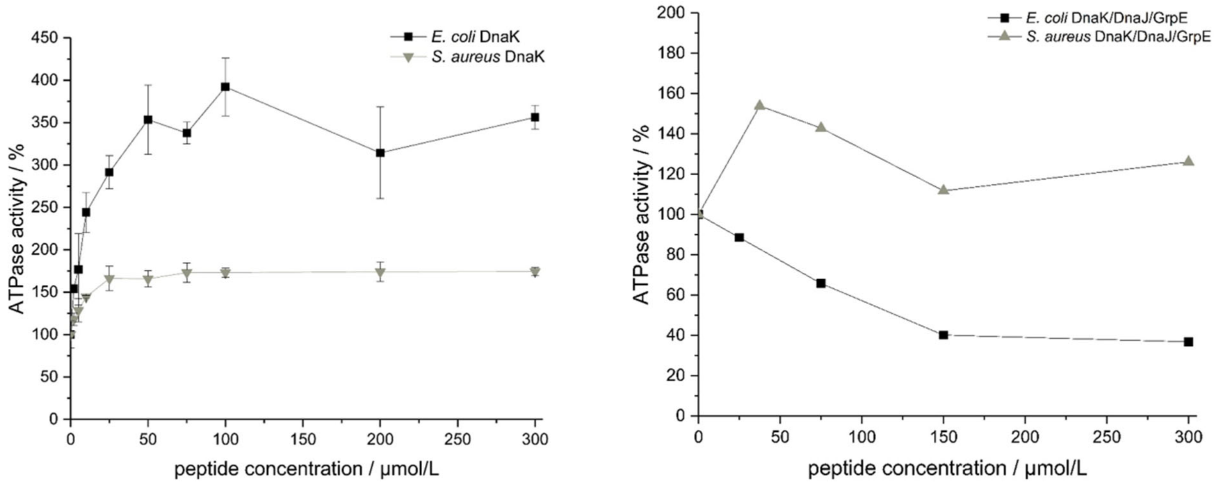

Figure S5: ATPase activity determined for an ARV-1502 dilution series in the absence (left) and presence of co-chaperones DnaJ and GrpE (right).

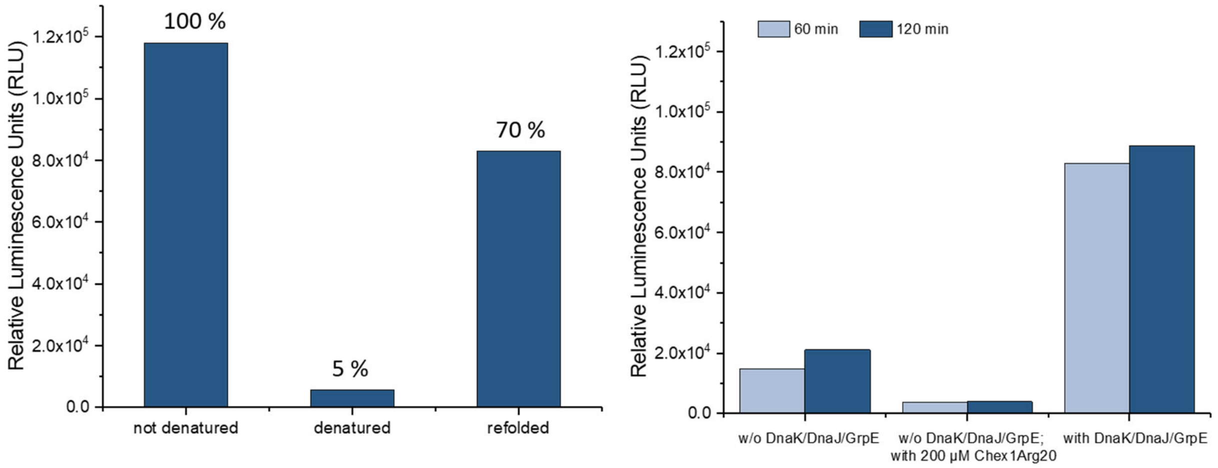

Figure S6: Relative luminescence intensity obtained for the control samples of the denatured luciferase refolding activity assay. Luminescence obtained for firefly luciferase before and after denaturation and after refolding by the chaperone system (left). The refolding of denatured luciferase was also studied in the absence of chaperones after incubation periods of one and two hours (right).

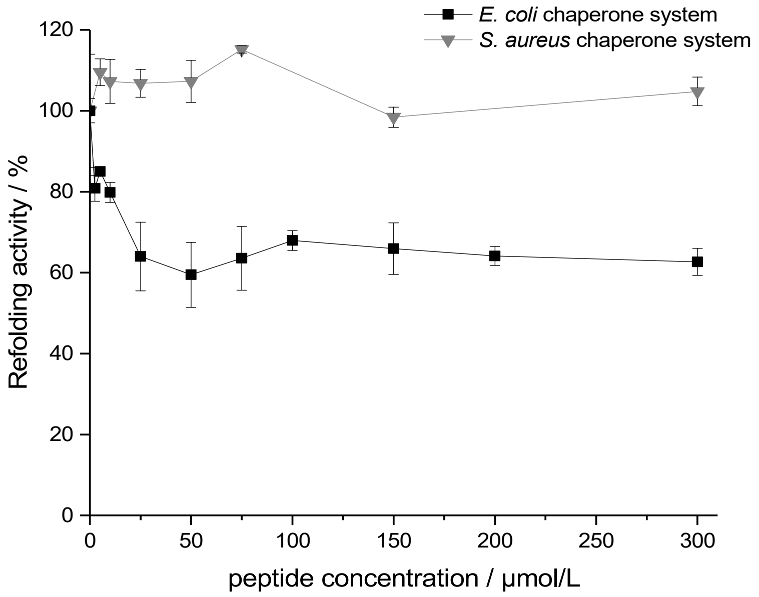

Figure S7: Refolding activity of *E. coli* and *S. aureus* chaperone system after incubation with ARV-1502 at different concentrations.

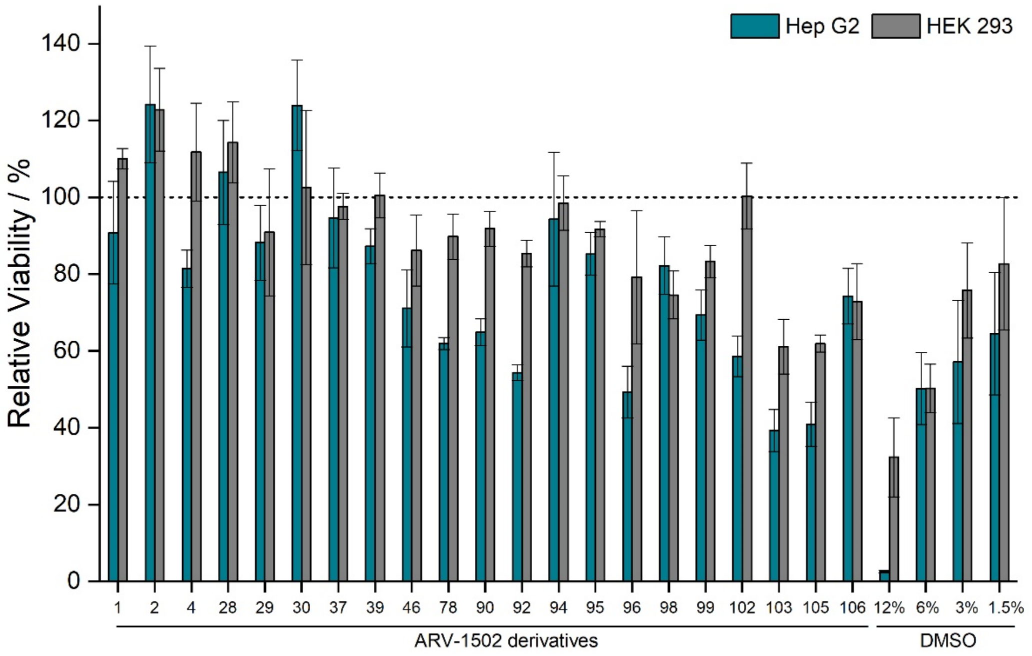

Figure S8: Relative viability of HepG2 and HEK293 cells after incubation with the most active peptides (MIC ≤ 16 µg/mL) for cytotoxicity testing. For each cell line, peptides were tested in three replicates once. As a control a dilution series of DMSO was added. Results were normalized to untreated cell (incubated with 12 % PBS).

REFERENCES

1. Zhang J-H, Chung TD, Oldenburg KR. A simple statistical parameter for use in evaluation and validation of high throughput screening assay. *J Biomol Screen* (1999):67–73.
